# Genetics, Receptor Binding, Replication, and Mammalian Transmission of H4 Avian Influenza Viruses Isolated from Live Poultry Markets in China

**DOI:** 10.1128/JVI.02692-15

**Published:** 2016-01-15

**Authors:** Libin Liang, Guohua Deng, Jianzhong Shi, Shuai Wang, Qianyi Zhang, Huihui Kong, Chunyang Gu, Yuntao Guan, Yasuo Suzuki, Yanbing Li, Yongping Jiang, Guobin Tian, Liling Liu, Chengjun Li, Hualan Chen

**Affiliations:** a State Key Laboratory of Veterinary Biotechnology, Harbin Veterinary Research Institute, Chinese Academy of Agricultural Sciences, Harbin, China; b College of Life and Health Sciences, Chubu University, Aichi, Japan

## Abstract

H4 avian influenza virus (AIV) is one of the most prevalent influenza virus subtypes in the world. However, whether H4 AIVs pose a threat to public health remains largely unclear. Here, we analyzed the phylogenetic relationships, receptor binding properties, replication, and transmissibility in mammals of H4 AIVs isolated from live poultry markets in China between 2009 and 2012. Genomic sequence analysis of 36 representative H4 viruses revealed 32 different genotypes, indicating that these viruses are undergoing complex and frequent reassortment events. All 32 viruses tested could replicate in the respiratory organs of infected mice without prior adaptation. Receptor binding analysis demonstrated that the H4 AIVs bound to α-2,6-linked glycans, although they retained the binding preference for α-2,3-linked glycans. When we tested the direct-contact transmission of 10 H4 viruses in guinea pigs, we found that three viruses did not transmit to any of the contact animals, one virus transmitted to one of three contact animals, and six viruses transmitted to all three contact animals. When we further tested the respiratory droplet transmissibility of four of the viruses that transmitted efficiently via direct contact, we found that three of them could transmit to one or two of the five exposed animals. Our study demonstrates that the current circulating H4 AIVs can infect, replicate in, and transmit to mammalian hosts, thereby posing a potential threat to human health. These findings emphasize the continual need for enhanced surveillance of H4 AIVs.

**IMPORTANCE** Numerous surveillance studies have documented the wide distribution of H4 AIVs throughout the world, yet the biological properties of H4 viruses have not been well studied. In this study, we found that multiple genotypes of H4 viruses are cocirculating in the live poultry markets of China and that H4 viruses can replicate in mice, possess human-type receptor binding specificity, and transmit between guinea pigs via direct contact. Strikingly, some H4 strains also can transmit via respiratory droplet, albeit with limited efficiency. These results clearly show the potential threat posed by H4 viruses to public health.

## INTRODUCTION

The influenza A virus genome comprises eight segments: basic polymerase 2 (PB2), basic polymerase 1 (PB1), acidic polymerase (PA), hemagglutinin (HA), nucleoprotein (NP), neuraminidase (NA), matrix (M), and nonstructural (NS) gene. On the basis of differences in the antigenicity of the two surface glycoproteins, HA and NA, influenza A viruses are categorized into different subtypes. Currently, 18 HA subtypes and 11 NA subtypes have been identified (1, 2). All of these subtypes were identified initially from avian species with the exception of H17N10 and H18N11, which were recently found in bats (1, 2).

Influenza pandemics occur when viruses bearing a novel HA protein are introduced into the human population and transmit efficiently among humans. Pandemic viruses emerge either by direct adaptation of an avian virus in a mammalian host, as occurred with the 1918 H1N1 pandemic (3), or by reassortment between human-, avian-, and even swine-origin viruses, as was the case with the emergence of the 1957 H2N2, 1968 H3N2, and 2009 swine-origin H1N1 pandemic viruses (4–7). Although these four human influenza pandemics all were caused by viruses of the H1, H2, or H3 subtypes, it would not be surprising for an influenza pandemic to be caused by a virus with a different HA subtype, because influenza viruses possess the ability to continuously evolve through mutation and reassortment in nature. Thus, the multiple subtypes of AIVs circulating in nature are a threat to public health and may have the potential to cause the next human influenza pandemic. The H5N1 highly pathogenic influenza viruses have spread to poultry and wild birds in over 60 countries (8–10) and sporadically infect humans, resulting in 449 deaths among 844 laboratory-confirmed human cases (http://www.who.int/). Several studies have demonstrated the transmission of H5N1 viruses among guinea pigs and ferrets via respiratory droplet after the acquisition of specific mutations or reassortment with human influenza viruses (11–14). In February 2013, a new H7N9 avian influenza virus emerged in China (15), and as of 15 October 2015, this virus has claimed the lives of 275 people among 679 confirmed cases of infection (http://www.who.int/). Transmission studies have shown that some human H7N9 strains have acquired partial or complete respiratory droplet transmissibility among ferrets (16–20). Since its first isolation in Wisconsin in 1966 (21), the H9N2 virus has been circulating widely in the world (22) and has sporadically caused human infections (23). In 2009, Sorrell et al. demonstrated that an experimentally generated avian-human reassortant H9N2 virus, possessing the HA and NA genes of an early H9N2 isolate and the six internal genes of a human H3N2 virus, transmitted among ferrets via respiratory droplet after acquiring adaptive mutations upon 10 serial passages in ferrets (24). We recently studied the contemporary avian H9N2 viruses and found that some of the natural H9N2 strains have acquired respiratory droplet transmissibility in ferrets (25). In addition to the threats posed by the H5, H7, and H9 AIVs, other subtypes of AIVs, including H6N1 and H10N8 viruses, also can cause human infections and even deaths (26, 27). The H6 AIVs can infect mice and ferrets without prior adaptation, and some can transmit efficiently by direct contact among guinea pigs (28, 29). The H10 viruses have partially acquired human-type receptor binding ability and can cause up to 22.5% body weight loss in mice (30, 31). Taken together, these findings indicate that AIVs of various HA subtypes represent a huge potential threat to public health.

The H4 AIV is also widely circulating in the world. It has been detected frequently from wild and domestic avian species in surveillance studies conducted in Asian, European, and North American countries since it was first isolated from a duck in Czechoslovakia in 1956 (32–48). It can infect mice directly without prior adaptation (32, 49). In a recent study, Bui et al. showed that H4N8 AIVs isolated from shorebirds in eastern Hokkaido can cause severe respiratory disease and even death in some mice (35). Currently, three swine infection cases of H4 AIVs under natural conditions have been reported in Canada and China (43, 50, 51). In addition, increasing seroepidemiologic evidence demonstrated that H4 AIVs can infect pigs in China (52, 53) and even poultry farmers and workers in the United States and Lebanon (54, 55).

So far, more than 1,800 H4 HA sequences have been deposited in the NCBI's Influenza Virus Sequence Database. These viruses were derived mostly from wild bird samples, but some are from samples collected from live poultry markets. Although surveillance studies have shown that the various subtypes of H4 AIVs are widely circulating throughout the world, the biological properties of the H4 viruses have not been well studied. Here, we fully analyzed the phylogenetic relationship, receptor binding specificity, replicative ability, and transmissibility in mammals of a series of H4 viruses that were isolated from samples collected in the live poultry markets of China between 2009 and 2012. Our results clearly reveal the public health concern represented by the H4 viruses.

## MATERIALS AND METHODS

### Ethics statements and facility.

The present study was carried out in strict accordance with the recommendations in the Guide for the Care and Use of Laboratory Animals of the Ministry of Science and Technology of the People's Republic of China. The protocol was approved by the Committee on the Ethics of Animal Experiments of the Harbin Veterinary Research Institute (HVRI) of the Chinese Academy of Agricultural Sciences (CAAS) (approval numbers BRDW-XBS-09S for mice and BRDW-TS-09S for guinea pigs).

### Virus isolation and identification.

The H4 subtype AIVs used in this study were isolated during routine surveillance between 2009 and 2012 from samples collected from chickens, ducks, or geese housed in live poultry markets in China. Cloacal and tracheal swabs from the same bird were put into one collection tube with 1 ml of phosphate-buffered saline (PBS; containing 1,000 U/ml penicillin and 2,000 μg/ml streptomycin) and were counted as one sample. All samples were individually inoculated into 10-day-old embryonated chicken eggs for 48 h at 37°C. The allantoic fluid was collected and tested for hemagglutination activity with 0.5% chicken red blood cells (cRBCs). HA subtypes were identified by using the hemagglutination inhibition (HI) test and by sequencing. NA subtypes were determined by means of direct sequencing. Virus stocks were propagated in Madin-Darby canine kidney (MDCK) cells in Dulbecco's modified Eagle's medium (DMEM; Corning, New York). All virus isolation procedures were conducted in a biosafety level 3 (BSL3) facility approved for such use by the Ministry of Agriculture, China.

### Sequence analyses.

A total of 36 H4 isolates were used for genetic and phylogenetic analyses in this study. RNA extraction was performed by using a QIAamp viral RNA (vRNA) minikit (Qiagen, Valencia, CA) according to the manufacturer's instructions. cDNA was synthesized from vRNA by reverse transcription with the Uni12 primer (56) and amplified by PCR with primers complementary to the conserved promoter and noncoding region of each gene segment (primer sequences are available upon request). Sequencing was performed using the BigDye Terminator cycle sequencing kit (Applied Biosystems, Foster City, CA) and run on an ABI 3500xL genetic analyzer (Applied Biosystems). The nucleotide sequences were edited by using the SeqMan module of the DNAStar package. Phylogenetic analysis was performed by using the MEGA5.0 software package, implementing the neighbor-joining method. The tree topology was evaluated by 1,000 bootstrap analyses.

To investigate whether the viruses recovered from guinea pigs had acquired mutations, viral RNA was extracted from nasal washes and reverse transcribed into cDNA as described above. All eight gene segments were amplified by PCR and inserted into the pEASY-Blunt Zero cloning vector (Transgen, Beijing, China). The sequences of 15 positive clones of each construct were examined for potential mutations in the viral genome.

### Antigenic analyses.

Antigenic analyses were performed by means of HI tests with chicken antisera generated against the viruses indicated in Table 2. To generate the antisera, 6-week-old specific-pathogen-free (SPF) chickens were inoculated with 10^6.0^ 50% egg infective doses (EID_50_) of test virus, and sera were collected at 3 weeks postinoculation (p.i.). The antisera were titrated for the presence of HI antibody with 0.5% cRBCs. The antigens used were the stocks of the wild-type viruses. Titers were recorded as the inverse of the highest antibody dilution that inhibited 8 agglutinating units of virus.

### Receptor binding analysis using a solid-phase binding assay.

Receptor binding specificity was analyzed by use of a solid-phase binding assay as described previously (12, 31, 57), using two different glycopolymers: α-2,3-siaylglycopolymer [Neu5Acα2-3Galβ1-4GlcNAcβ1-pAP (para-aminophenyl)-alpha-polyglutamic acid (α-PGA)] and α-2,6-sialylglycopolymer [Neu5Acα2-6Galβ1-4GlcNAcβ1-pAP (para-aminophenyl)-alpha-polyglutamic acid (α-PGA)]. For the 10 wild-type H4 viruses selected in this study and the HA mutants of A/duck/Jiangxi/S21055/12 (DK/Jiangxi/S21055/12, H4N2) virus described below, chicken antiserum against DK/Jiangxi/S21055/12 (H4N2) virus was used as the primary antibody; for the A/duck/Czechoslovakia/1956 (DK/Czechoslovakia/56, H4N6) virus, chicken antiserum against the same virus was used as the primary antibody. A horseradish peroxidase (HRP)-conjugated goat-anti-chicken antibody (Sigma-Aldrich, St. Louis, MO) was used as the secondary antibody. Absorbance was determined at a wavelength of 490 nm. Two viruses, A/chicken/Hebei/3/13 (CK/Hebei/3/13, H5N2) and A/Sichuan/1/09 (Sichuan/1/09, H1N1), that bind exclusively to α-2,3- and α-2,6-linked sialic acid (SA) receptors (31), respectively, were used as controls.

### Generation of mutant DK/Jiangxi/S21055/12 (H4N2) viruses.

The eight gene segments of DK/Jiangxi/S21055/12 (H4N2) were inserted into the bidirectional transcription vector pBD as described previously (58). Mutations were introduced into the HA gene of DK/Jiangxi/S21055/12 (H4N2) virus by site-directed mutagenesis with the QuikChange mutagenesis kit (Stratagene, La Jolla, CA) according to the manufacturer's protocol. The primer sequences are available upon request. All of the constructs were completely sequenced to ensure the absence of unwanted mutations. The HA mutant viruses were generated by using reverse genetics as described previously (58). The identity of the mutant viruses was confirmed by completely sequencing the viral genome.

### Studies of mice.

Groups of eight 6-week-old female BALB/c mice (Vital River Laboratories, Beijing, China) were lightly anesthetized with CO_2_ and inoculated intranasally (i.n.) with 10^6.0^ EID_50_ of different H4 influenza viruses in a volume of 50 μl. Control mice were inoculated with 50 μl of PBS. On day 3 p.i., three of the eight inoculated mice in each group were euthanized, and their organs, including lungs, nasal turbinates, kidneys, spleen, and brain, were collected for virus titration in eggs. The virus titers were calculated by the method of Reed and Muench. The remaining five mice in each group were monitored daily for weight loss and survival for 14 days.

### Studies in guinea pigs.

Hartley strain female guinea pigs weighing 300 to 350 g (Vital River Laboratories, Beijing, China) that were serologically negative for influenza viruses were used in this study. Ketamine (20 mg/kg of body weight) and xylazine (1 mg/kg) were used to anesthetize the animals by intramuscular injection. To determine the replication ability of the influenza viruses, groups of two guinea pigs were anesthetized and inoculated i.n. with 10^6.0^ EID_50_ of test virus in a 300-μl volume (150 μl per nostril). The animals were euthanized on day 3 p.i. to collect nasal washes, trachea, and lungs for virus titration in eggs.

For the direct-contact transmission experiments, groups of three guinea pigs housed in a cage placed inside an isolator were inoculated i.n. with 10^6.0^ EID_50_ of test virus. Twenty-four hours later, three naive animals were introduced into the same cage. Nasal washes were collected at 2-day intervals, beginning on day 2 p.i. (1 day after contact). Viruses in the nasal washes were titrated in eggs.

For the respiratory droplet transmission experiment, groups of five guinea pigs housed in a cage placed inside an isolator were inoculated i.n. with 10^6.0^ EID_50_ of test virus. Twenty-four hours later, five naive guinea pigs were placed in an adjacent cage (4 cm away), separated by a double-layered net divider. The ambient conditions for these studies were set at 20 to 22°C and 30 to 40% relative humidity. The airflow in the isolator was horizontal with a speed of 0.1 m/s, and the airflow direction was from the inoculated animals to the exposed animals. Nasal washes were collected at 2-day intervals, beginning on day 2 p.i. (1 day postexposure [p.e.]) and titrated in eggs. Sera were collected from all animals on day 21 p.i. for HI antibody detection. To prevent inadvertent physical transmission of virus by the investigators, the exposed guinea pigs were always handled first, and gloves, implements, and napkins on the work surface were changed between animals.

### HI antibody detection in guinea pig sera.

Guinea pig sera were treated with a receptor-destroying enzyme from Vibrio cholerae (Denka Seiken Co., Ltd., Tokyo, Japan) at 37°C for 24 h before being tested for the presence of HI antibody with 0.5% cRBCs. The antigens used were the stocks of wild-type viruses. Titers were recorded as the inverse of the highest antibody dilution that inhibited 8 agglutinating units of virus.

### Nucleotide sequence accession numbers.

The nucleotide sequences of the 36 H4 viruses determined in this study have been deposited in GenBank under accession numbers KU160800 to KU161087.

## RESULTS

### Molecular characterization of H4 AIVs.

During our routine surveillance of AIVs between January 2009 and December 2012, we collected samples from live poultry markets and isolated viruses from 10-day-old embryonated chicken eggs. To better understand the genetic relationships among H4 AIVs, we sequenced the complete genomes of 36 H4 viruses, representing H4 isolates from different hosts (chickens, ducks, or geese), different subtypes (12 H4N2, one H4N3, 19 H4N6, and four H4N8 viruses), and different provinces in China (Anhui, Chongqing, Fujian, Guangdong, Guangxi, Guizhou, Henan, Hubei, Hunan, Jiangsu, Jiangxi, Shandong, Sichuan, and Zhejiang) (Table 1). All of these H4 viruses possessed a single basic amino acid (arginine) in the HA cleavage site, the signature of low-pathogenicity AIVs (59). The receptor binding site of influenza virus HA is formed by the 190-helix, 130-loop, and 220-loop at its globular head (60). The HA receptor binding site of all 36 viruses in this study was highly conserved, containing 98Y, 134G, 135K, 136S, 137G, 138A, 153W, 155V, 183H, 190E, 194L, 195Y, 224R, 225G, 226Q, 227S, 228G, and 229R (H3 numbering, which is used throughout this work). None of these residues have been reported to be involved in the recognition of human-type receptors. All of these H4 AIVs had five conserved potential glycosylation sites in HA at positions 6 to 8, 22 to 24, 165 to 167, 296 to 298, and 483 to 485, respectively. The NA gene of A/duck/Hubei/S2227/12 (DK/Hubei/S2227/12, H4N2) had a 9-nucleotide deletion at positions 186 to 194 that resulted in the loss of three amino acids at positions 63 to 65. There were no other deletions in the NA genes of any of the other viruses.

**TABLE 1:** Genotypes and replication of H4 avian influenza viruses in mice

Virus	Group of each gene segment in the phylogenetic tree								Genotype^*a*^	Replication of H4 AIVs in mice^*b*^ (log_10_ EID_50/_ml)		Maximum body wt loss^*c*^ (%)
	HA	NA	PB2	PB1	PA	NP	M	NS		Nasal turbinate	Lung	
DK/Jiangxi/S3261/09 (H4N2)	2	1	3	6	6	6	1	4	A1	4.2 ± 0.3	3.6 ± 0.2	−1.9
DK/Guangdong/S1469/10 (H4N2)	2	1	3	6	6	6	3	3	A2	3.2 ± 0.5	2.8 ± 0.4	−3.0
DK/Guangdong/S4040/11 (H4N2)	2	1	3	6	7	7	2	3	A3	3.7 ± 0.1	5.5 ± 0.2	0.0
DK/Hunan/S2046/11 (H4N2)	1	1	5	2	2	10	4	4	A4	2.3 ± 0.2	4.0 ± 0.5	−4.8
DK/Sichuan/S4202/11 (H4N2)	1	2	5	1	5	10	1	1	A5	1.2 ± 0.3	3.5 ± 0.2	−4.0
DK/Guangdong/S1123/12 (H4N2)	2	1	3	6	6	6	5	3	A6	ND	ND	ND
GS/Guangdong/S1780/12 (H4N2)	2	1	3	6	6	6	5	3	A6	4.4 ± 0.7	4.0 ± 0.5	−7.3
DK/Hubei/S2213/12 (H4N2)	1	1	2	1	7	3	3	4	A7	5.0 ± 0.3	5.6 ± 0.1	−6.4
CK/Hubei/S2227/12 (H4N2)	1	3	4	7	3	11	3	2	A8	3.5 ± 0.5	5.0 ± 0.4	−4.5
DK/Jiangxi/S21046/12 (H4N2)	1	1	6	6	2	7	2	4	A9	3.2 ± 0.3	3.8 ± 0.5	−4.0
DK/Jiangxi/S21055/12 (H4N2)	1	1	2	1	2	7	2	4	A10	4.0 ± 0.5	5.2 ± 0.6	−4.5
CK/Shandong/S2510/12 (H4N2)	1	2	5	1	5	4	1	4	A11	4.8 ± 0.5	4.8 ± 0.5	−6.0
DK/Fujian/S1487/09 (H4N3)	3	1	1	6	1	8	1	4	B1	2.3 ± 0.4	4.5 ± 0.3	0.0
DK/Anhui/S4155/09 (H4N6)	1	2	5	1	5	1	1	4	C1	<	2.1 ± 0.4	−1.2
DK/Hunan/S1012/09 (H4N6)	2	1	5	1	7	4	4	4	C2	3.6 ± 0.5	4.0 ± 0.7	−1.5
DK/Henan/S4179/09 (H4N6)	1	2	1	1	6	8	1	4	C3	2.1 ± 0.4	4.0 ± 0.3	0.0
CK/Hunan/S1248/10 (H4N6)	1	1	2	1	5	1	1	4	C4	2.3 ± 0.2	4.3 ± 0.4	−6.0
CK/Hunan/S1267/10 (H4N6)	1	1	2	1	5	1	1	4	C4	ND	ND	ND
DK/Guangxi/S1211/10 (H4N6)	1	2	5	1	7	1	1	1	C5	4.0 ± 0.5	4.8 ± 0.7	−3.0
DK/Guangxi/S1107/10 (H4N6)	1	2	5	1	7	1	1	1	C5	ND	ND	ND
DK/Guizhou/S1167/10 (H4N6)	1	2	5	1	7	8	1	1	C6	1.3 ± 0.4	3.5 ± 0.5	0.0
DK/Henan/S1091/10 (H4N6)	1	1	5	1	7	4	1	4	C7	1.5 ± 0.2	4.8 ± 0.5	−4.0
DK/Hunan/S1166/10 (H4N6)	1	2	1	3	7	4	5	1	C8	2.0 ± 0.5	4.2 ± 0.2	−13
GS/Jiangsu/S2433/11 (H4N6)	1	2	2	1	7	4	1	4	C9	1.7 ± 0.5	5.3 ± 0.2	−6.4
DK/Jiangsu/S2447/11 (H4N6)	1	2	2	1	7	4	1	4	C9	ND	ND	ND
DK/Zhejiang/S2088/11 (H4N6)	1	2	6	4	2	7	2	4	C10	4.5 ± 0.3	6.0 ± 0.3	−7.7
DK/Guangxi/S4312/11 (H4N6)	1	2	2	1	7	1	1	1	C11	2.8 ± 0.5	4.0 ± 0.3	−8.5
DK/Anhui/S2193/12 (H4N6)	1	2	5	4	5	1	3	4	C12	3.6 ± 0.6	4.0 ± 0.5	0.0
DK/Fujian/S2169/12 (H4N6)	1	2	6	5	2	7	2	4	C13	3.5 ± 0.6	4.0 ± 0.3	−1.0
DK/Guangxi/S2090/12 (H4N6)	1	2	5	1	4	8	1	4	C14	3.0 ± 0.7	3.5 ± 0.3	0.0
DK/Jiangxi/S2443/12 (H4N6)	1	1	1	1	5	2	4	4	C15	2.3 ± 0.3	4.6 ± 0.2	0.0
DK/Zhejiang/S2235/12 (H4N6)	1	1	6	5	2	7	2	4	C16	3.5 ± 0.5	3.8 ± 0.7	0.0
CK/Guangdong/S1010/10 (H4N8)	2	1	3	6	6	5	3	4	D1	3.0 ± 0.5	4.0 ± 0.5	−6.7
GS/Hunan/S2466/11 (H4N8)	2	3	1	3	5	9	1	4	D2	2.5 ± 0.2	5.0 ± 0.5	−2.0
DK/Chongqing/S2086/12 (H4N8)	1	4	5	1	5	1	1	4	D3	4.2 ± 0.3	6.0 ± 0.5	−4.0
DK/Hubei/S2114/12 (H4N8)	1	2	2	6	7	4	4	4	D4	<	4.8 ± 0.3	0.0

a Genotypes were defined on the basis of the gene phylogenies of the eight viral genes shown in Fig. 1 and 2 (also see Fig. S1 in the supplemental material).

b Six-week-old BALB/c mice were inoculated i.n. with 10^6.0^ EID_50_ of each virus in a 50-μl volume. Three mice from each group were euthanized on day 3 p.i., and virus titers were determined in samples of brain, spleen, kidney, nasal turbinate, and lung in eggs. Because virus was not detected in the brain, spleen, or kidneys of any infected mice, only the virus titers in the nasal turbinates and lungs are shown. ND, not done; <, virus was not detected from the undiluted sample.

c The maximum body weight loss was calculated by comparing the lowest body weight during the 14-day observation period to the initial body weight of the infected mice. A value of 0.0 indicates that the infected mice did not show body weight loss during the observation period. ND, not done.

Several residues in PB2, especially 627K, 701N, and 271A, are known to play an important role in the virulence and transmission of influenza viruses in mammals (58, 59, 61–64). None of these known mutations were observed in the H4 AIVs in this study. In the M2 protein, mutations involved in resistance to the anti-influenza drugs amantadine and rimantadine were detected in several strains: V27I in three viruses (A/duck/Guangdong/S1469/10 [DK/Guangdong/S1469/10, H4N2], A/duck/Hubei/S2213/12 [DK/Hubei/S2213/12, H4N2], and DK/Hubei/S2227/12 [H4N2]), S31N in A/duck/Anhui/S2193/12 (DK/Anhui/S2193/12, H4N6), and the double mutation of L26F and V27I in A/chicken/Guangdong/S1010/10 (CK/Guangdong/S1010/10, H4N8). There were no deletions in the NS1 protein of any of the H4 AIVs sequenced, with the exception of DK/Hubei/S2227/12 (H4N2), which had a 13-amino-acid deletion at positions 218 to 230. All of these H4 viruses had a full-length PB1-F2 protein of 90 amino acids, and none of them had the N66S mutation, which has been shown to increase the virulence of AIVs in mammalian hosts (65).

### Phylogenetic analysis.

The phylogenetic tree of the H4 HA genes was separated into the North American lineage and the Eurasian lineage. All 36 H4 HA genes in this study clustered into the Eurasian lineage, with nucleotide identities of between 84.8% and 100%, and could be divided into 3 groups (Fig. 1A). The intragroup homology was over 95%, whereas the intergroup homology was less than 92.2%. Group 1 contained 27 viruses, including 7 H4N2, 18 H4N6, and 2 H4N8 viruses, which were isolated from different poultry species across 13 provinces. All five HA genes of the H4 viruses isolated from Guangdong province clustered in group 2, and the HA genes of two viruses isolated from Hunan province and one from Jiangxi province also clustered in this group. Group 3 had only one virus, A/duck/Fujian/S1487/09 (DK/Fujian/S1487/09, H4N3). Its HA gene was most closely related to that of A/duck/Poyang Lake/FB13/2007 (H4N6) in GenBank, with 96% nucleotide sequence identity.

**FIG 1:**
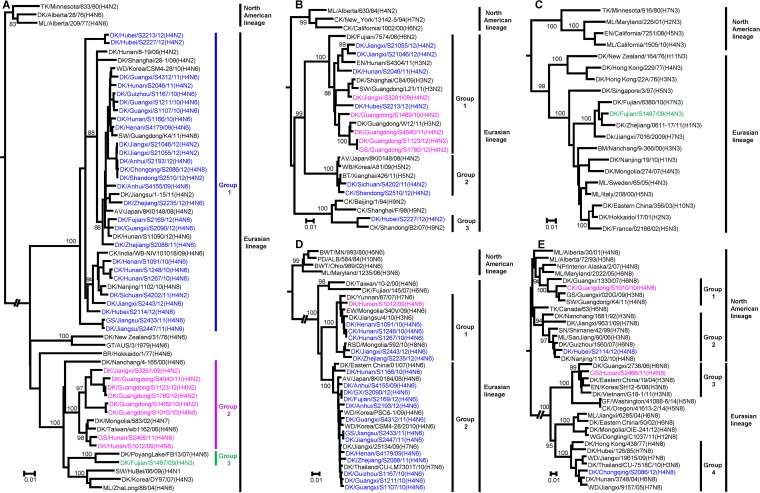
Phylogenetic analyses of the HA and NA genes of H4 avian influenza viruses isolated from live poultry markets in China between 2009 and 2012. Phylogenetic trees were generated by using the neighbor-joining method and the MEGA5.0 software package. Neighbor-joining bootstrap values of ≥80 are shown at the major nodes of the phylogenetic trees. The regions of nucleotide sequences used for the phylogenetic analyses were the following: H4 HA, 20 to 1714; N2 NA, 20 to 1357; N3 NA, 19 to 1425; N6 NA, 40 to 1421; and N8 NA, 21 to 1433. The phylogenetic tree of the H4 HA genes was rooted to A/swine/Ontario/01911-1/99 (H4N6) (A), the N2 NA tree was rooted to A/blue-winged teal/Ohio/908/2002 (H3N2) (B), the N3 NA tree was rooted to A/black headed gull/Mongolia/1756/2006 (H16N3) (C), the N6 NA tree was rooted to A/duck/Wisconsin/480/1979 (H12N6) (D), and the N8 NA tree was rooted to A/mallard/Ohio/123/1989 (H6N8) (E). The viruses sequenced in this study are colored in the phylogenetic tree based on the phylogenetic classification of their HA genes: blue, magenta, and green for groups 1, 2, and 3, respectively. Abbreviations: AV, avian; BR, budgerigar; BWT, blue-winged teal; CK, chicken; DK, duck; EN, environment; EW, Eurasian wigeon; GF, gyrfalcon; GS, goose; GT, gray teal; MD, migratory duck; ML, mallard; NP, northern pintail; PD, pintail duck; RSD, ruddy shelduck; SN, swan; SW, swine; TK, turkey; WB, wild bird; WD, wild duck; WG, wild goose.

Of the 36 H4 viruses sequenced in this study, 12 possessed an NA gene of the N2 subtype. These viruses were phylogenetically diverse and formed three groups (Fig. 1B). The nine N2 NA genes in group 1 were closely related to the NA genes of H3N2 AIVs isolated in southern China. Group 2 included two N2 NA genes, A/duck/Sichuan/S4202/11 (DK/Sichuan/S4202/11, H4N2) and A/chicken/Shandong/S2510/12 (CK/Shandong/S2510/12, H4N2). The NA gene of DK/Hubei/S2227/12 (H4N2) in group 3 formed a cluster with the NA genes of the H9N2 viruses. The only N3 NA gene, from DK/Fujian/S1487/09 (H4N3) virus, belonged to the Eurasian lineage and was most closely related to the NA genes of H1N3 and H7N3 AIVs isolated in Zhejiang and Fujian provinces (Fig. 1C). The 19 N6 NA genes formed 2 groups, with 6 in group 1 and 13 in group 2 (Fig. 1D). The phylogenetic tree of the N8 NA genes was separated into the North American and Eurasian lineages (Fig. 1E). The N8 NA gene of the North American lineage was first introduced into China in 1992, as evidenced by the identification of A/duck/Nanchang/1681/92 (DK/Nanchang/1681/92, H3N8) from GenBank. In the present study, the N8 NA gene of CK/Guangdong/S1010/10 (H4N8) belonged to group 1 in the North American lineage, whereas the N8 NA gene of A/duck/Hubei/S2114/12 (DK/Hubei/S2114/12, H4N8) was a descendant of DK/Nanchang/1681/92 (H3N8) and belonged to group 2 in the North American lineage. In contrast, the other two N8 NA genes of A/goose/Hunan/S2466/11 (GS/Hunan/S2466/11, H4N8) and A/duck/Chongqing/S2086/12 (DK/Chongqing/S2086/12, H4N8) belonged to two different groups in the Eurasian lineage. Toward the end of 2014, a novel highly pathogenic H5N8 avian influenza virus spread and caused outbreaks in Canada and the United States (10). It was notable that this H5N8 virus possessed an NA gene derived from the Eurasian lineage and was phylogenetically distinct from the N8 NA genes of the North American lineage in this study.

**FIG 2:**
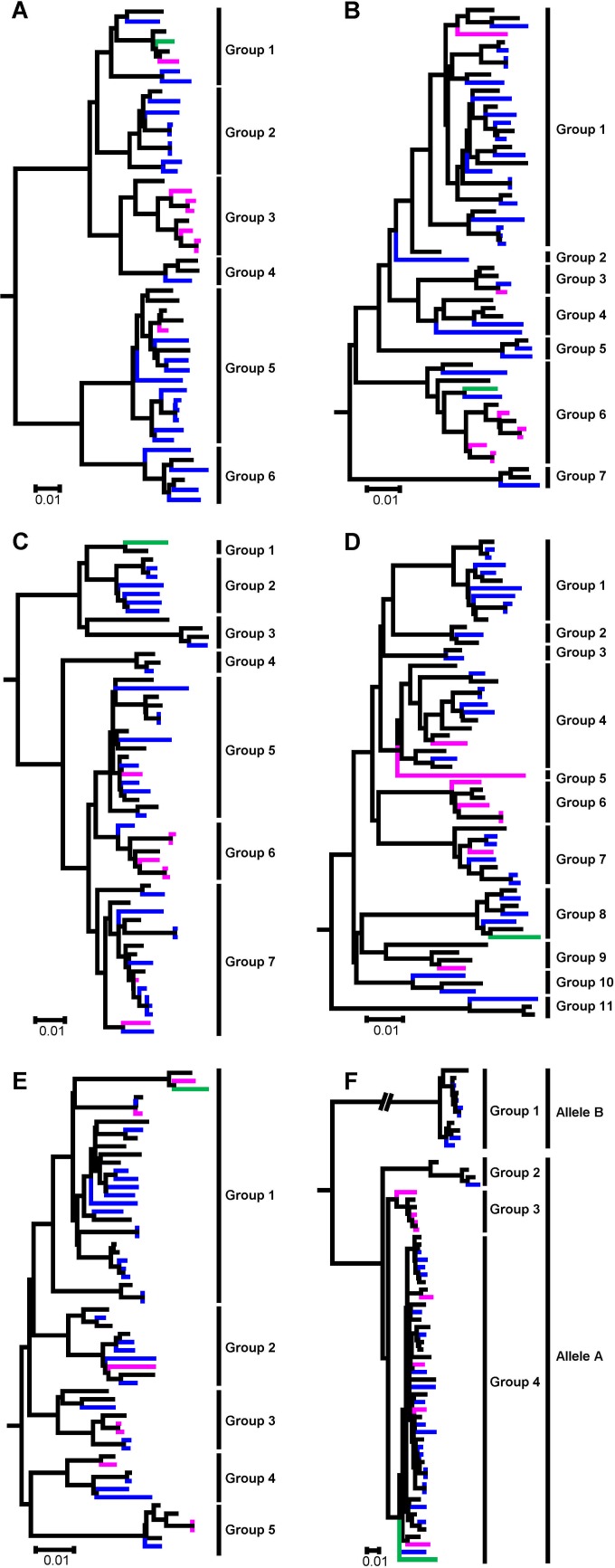
Phylogenetic analyses of the six internal genes of H4 avian influenza viruses isolated from live poultry markets in China between 2009 and 2012. Phylogenetic trees were generated by using the neighbor-joining method and the MEGA5.0 software package. The regions of nucleotide sequence used for the phylogenetic analyses were the following: PB2, 37 to 2289; PB1, 25 to 2233; PA, 25 to 2167; NP, 46 to 1526; M, 26 to 1007; and NS, 27 to 859. The phylogenetic trees of PB2 (A), PB1 (B), PA (C), NP (D), M (E), and NS (F) all were rooted to A/equine/Prague/1/1956 (H7N7). The phylogenetic branches of viruses sequenced in this study are colored in the phylogenetic tree based on the colors of their HA genes: blue, magenta, and green for groups 1, 2, and 3, respectively. Larger versions of these phylogenetic trees are provided in Fig. S1A to F in the supplemental material.

Phylogenetic analysis of the 36 PB2 genes revealed that they formed six distinct groups (Fig. 2A; also see Fig. S1A in the supplemental material). The PB2 genes shared nucleotide sequence identities of less than 94.1% among the different groups. The PB1 genes of the 36 H4 AIVs formed seven groups in the phylogenetic tree (Fig. 2B; also see Fig. S1B). The nucleotide identities of the PB1 genes were less than 95.0% between the different groups. The PB1 gene in group 2, A/duck/Hunan/S2046/11 (DK/Hunan/S2046/11, H4N2), shared only 96.0% identity with the closest PB1 gene, A/goose/Eastern China/17/2010 (H6N6), in GenBank. The PB1 gene in group 7, DK/Hubei/S2227/12 (H4N2), shared less than 91.0% identity with the PB1 genes in the other groups. The PA genes of the 36 H4 AIVs clustered into seven groups in the phylogenetic tree and shared identities of less than 95.5% among the different groups (Fig. 2C; also see Fig. S1C). The NP genes of the 36 H4 AIVs were the most diverse, forming 11 distinct groups in the phylogenetic tree and sharing less than 95.2% identity among the different groups (Fig. 2D; also see Fig. S1D).

The M genes of the 36 viruses clustered into five groups with nucleotide sequence identities of less than 96.5% among the different groups (Fig. 2E; also see Fig. S1E in the supplemental material). Among these five groups, group 1 contained half of the 36 M genes. The NS genes of influenza A viruses were separated into two alleles, A and B (66). Six viruses (one H4N2 and five H4N6) in this study possessed NS genes of allele B (Fig. 2F; also see Fig. S1F). The other 30 viruses possessed NS genes of allele A and clustered into three groups. Among the three groups of allele A, the NS gene of DK/Hubei/S2227/12 (H4N2) belonged to group 2 and was a descendant of the A/chicken/Beijing/1/94 (CK/Beijing/1/94, H9N2)-like viruses. The four NS genes of H4N2 viruses isolated from Guangdong province clustered into group 3. The other 25 NS genes in group 4 contained multiple virus subtypes; this was the dominant group among the NS gene groups in this study.

On the basis of the phylogenetic diversity, the 36 H4 AIVs in this study were divided into 32 genotypes, including 11 genotypes for the 12 H4N2 viruses, one genotype for the only H4N3 virus, 16 genotypes for the 19 H4N6 viruses, and four genotypes for the four H4N8 viruses. Among these 32 genotypes, four genotypes (A6, C4, C5, and C9) included two viruses, while all of the other 28 genotypes each contained only one virus. These results indicate that complicated reassortments of H4 AIVs have occurred in the live poultry markets of China.

### Antigenic analysis.

Chicken antisera were generated against six H4 viruses, including three viruses of group 1, two viruses of group 2, and one virus of group 3 in the HA phylogenetic tree. To determine the antigenic variation of the H4 AIVs in this study, we performed an HI assay with 0.5% cRBCs. As shown in Table 2, there was less than an 8-fold difference between the HI titers of the six antisera against the homologous and heterologous viruses. It was notable that the antigenicity of the only virus in group 3, DK/Fujian/S1487/09 (H4N3), was different from the viruses in groups 1 and 2. These data suggest that the antigenic drift of H4 viruses circulating in China has occurred to some extent.

**TABLE 2:** Antigenic analysis of H4 avian influenza viruses isolated from live poultry markets in China

Virus (HA group)	HI antibody titer of antiserum against virus^*a*^ (HA group)					
	DK/Guizhou/S1167/10 (H4N6) (1)	DK/Jiangxi/S21046/12 (H4N2) (1)	GS/Jiangsu/S2433/11 (H4N6) (1)	DK/Guangdong/S1780/12 (H4N2) (2)	DK/Jiangxi/S3261/09 (H4N2) (2)	DK/Fujian/S1487/09 (H4N3) (3)
DK/Guizhou/S1167/2010 (H4N6) (1)	**512**	128	64	64	64	32
DK/Jiangxi/S21046/2012 (H4N2) (1)	512	**256**	128	128	128	64
GS/Jiangsu/S2433/2011 (H4N6) (1)	128	64	**128**	32	32	16
DK/Guangdong/S1780/2012 (H4N2) (2)	128	128	256	**128**	64	32
DK/Jiangxi/S3261/2009 (H4N2) (2)	512	128	64	128	**128**	64
DK/Fujian/S1487/2009 (H4N3) (3)	64	64	32	64	32	**128**

a Antiserum was generated by intranasally inoculating 6-week-old SPF chickens with 10^6.0^ EID_50_ of the indicated virus. The HA group indicates the phylogenetic classification of the virus HA gene in the phylogenetic tree. Homologous titers are shown in boldface.

### Replication of H4 AIVs in mice.

Of the 36 H4 viruses in this study, we selected one virus from each of the 32 genotypes to evaluate replicative ability and virulence in BALB/c mice. Groups of eight 6-week-old BALB/c mice were inoculated i.n. with 10^6.0^ EID_50_ of virus. Three mice in each group were euthanized on day 3 p.i., and their organs, including nasal turbinates, lungs, spleen, kidneys, and brain, were collected for virus titration in embryonated eggs; the remaining five mice were observed for 2 weeks for changes in body weight or disease signs. As shown in Table 1, all 32 viruses tested were detected in the lungs of mice, with mean titers ranging from 2.1 to 6.0 log_10_ EID_50_/ml; most of the viruses also were detected in the nasal turbinates of mice, with mean titers ranging from 1.2 to 5.0 log_10_ EID_50_/ml with the exception of A/duck/Anhui/S4155/09 (DK/Anhui/S4155/09, H4N6) and DK/Hubei/S2114/12 (H4N8), which were not detected in the nasal turbinates of any of the three inoculated mice. None of the viruses were detected in the other mouse organs, including spleen, kidneys, and brain. Body weight loss was observed in mice infected with some of the viruses tested within a range of not more than 13%. These results indicate that the H4 AIVs isolated in live poultry markets of China can replicate in the respiratory organs of mice, implying that H4 AIVs have the potential to directly infect mammalian hosts without prior adaptation.

### Receptor binding specificity of H4 AIVs.

**FIG 3:**
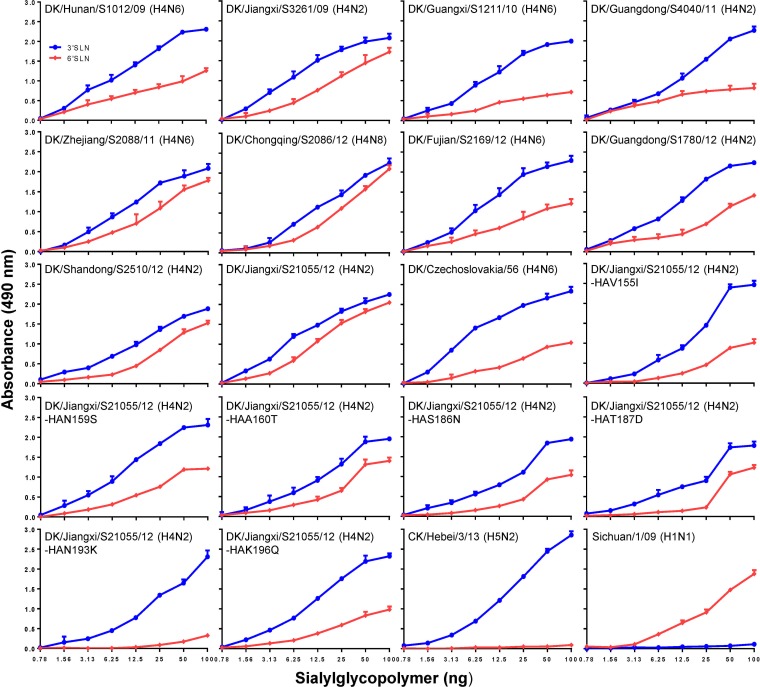
Characterization of the receptor binding properties of H4 avian influenza viruses. The H4 viruses and the HA mutants were compared on the basis of their ability to bind to sialylglycopolymers containing either α-2,3-linked (blue) or α-2,6-linked (red) glycans. Two viruses, CK/Hebei/3/13 (H5N2) and Sichuan/1/09 (H1N1), that bind exclusively to α-2,3- and α-2,6-linked sialic acid receptors, respectively, were used as controls. The data shown are the means from three replicates; the error bars indicate standard deviations.

The receptor binding preference of HA has important implications for influenza virus replication and transmission. Binding to α-2,6-linked sialic acids (SAs) is a prerequisite for an influenza virus to transmit efficiently among humans (64). The receptor binding site of all of the 36 H4 AIVs in this study was highly conserved. Therefore, we selected 10 viruses with different H4 subtypes that could replicate efficiently in mouse respiratory organs to investigate their receptor binding properties by using a solid-phase binding assay as described previously (12, 31, 57). We found that all 10 viruses tested could bind to the α-2,6-linked glycans, although their affinity for α-2,3-linked glycans was higher (Fig. 3). These results prompted us to investigate the receptor binding specificity of the earliest avian H4 isolate, DK/Czechoslovakia/56 (H4N6). We found that this prototype H4 virus also can bind to α-2,6-linked glycans. These data indicate that H4 AIVs intrinsically possess the ability to recognize human-type receptors.

### Asparagine at position 193 of HA is critical for the binding of H4 viruses to α-2,6-linked glycans.

All of the H4 AIVs in this study possessed identical amino acids at their receptor binding sites and could partially bind to human-type receptors. Therefore, we next sought to identify the key amino acids in H4 HA responsible for the human-type receptor binding ability. Because the receptor binding specificity of H4 AIVs has rarely been studied, we looked for potential HA amino acid sites that could confer partial human-type receptor binding affinity to H4 AIV by comparing H4 AIVs with H5N1 AIVs whose receptor binding preference has been intensively studied. Several amino acid mutations at or around the receptor binding sites in H5 HA have been shown to confer partial human-type receptor binding ability (e.g., I155T, S159N, T160A, N186K, D187G, K193R, and Q196H/Q196R) (14, 57, 67–69). Of note, the residues at these sites were different between the H4 viruses in this study and the H5N1 viruses that were used to study the role of these amino acid positions on receptor binding preference. We mutagenized these amino acid positions in the HA of DK/Jiangxi/S21055/12 (H4N2) to resemble the avian-type residues in H5 HA (i.e., V155I, N159S, A160T, S186N, T187D, N193K, and K196Q). By using plasmid-based reverse genetics, we generated mutant DK/Jiangxi/S21055/12 (H4N2) viruses harboring the individual mutations at these seven positions and named them according to the HA mutation they contained (e.g., DK/Jiangxi/S21055/12-HA V155I). Receptor binding analysis showed that all of these mutant viruses maintained strong binding to the α-2,3-linked glycans. In contrast, their binding affinity to the α-2,6-linked glycans was reduced compared to that of the wild-type DK/Jiangxi/S21055/12 (H4N2) virus (Fig. 3). Strikingly, the DK/Jiangxi/S21055/12-HA N193K variant displayed little to no binding to the α-2,6-linked glycans. These results indicate that the partial human-type receptor binding ability of the H4 AIV is determined by multiple residues at or around the HA receptor binding sites and that the asparagine residue at position 193 is essential for the binding of H4 viruses to the human-type receptor.

### Transmission of H4 AIVs in guinea pigs.

**FIG 4:**
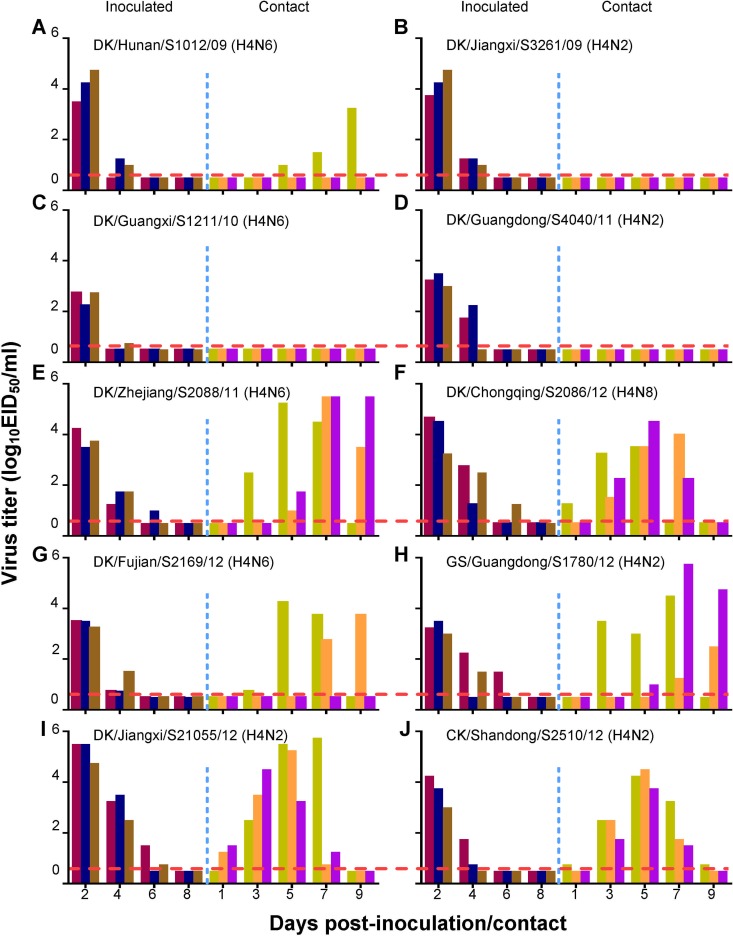
Direct-contact transmission of H4 avian influenza viruses in guinea pigs. Groups of three guinea pigs were inoculated i.n. with 10^6.0^ EID_50_ of test virus. Twenty-four hours later, three naive guinea pigs were introduced into the same cage. Nasal washes were collected at 2-day intervals. Each color bar represents the virus titer from an individual animal. The horizontal dashed red lines indicate the lower limit of detection.

**FIG 5:**
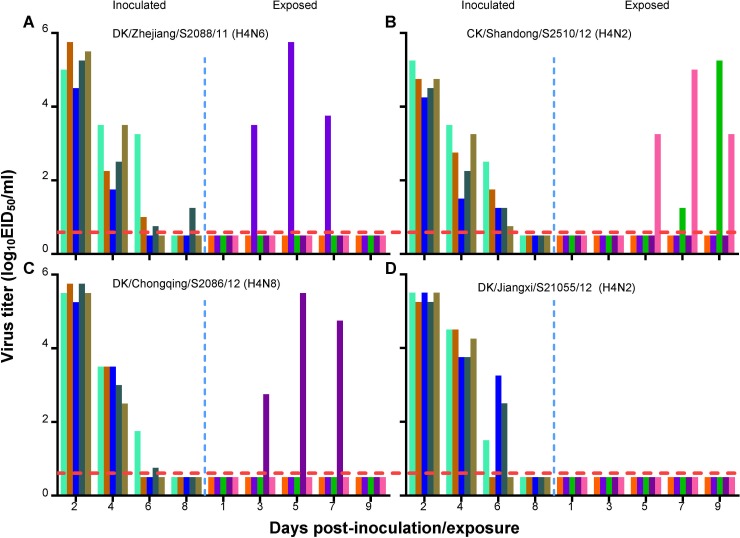
Respiratory droplet transmission of the H4 avian influenza viruses in guinea pigs. Groups of five guinea pigs were inoculated i.n. with 10^6.0^ EID_50_ of test virus. Twenty-four hours later, five naive guinea pigs were placed individually in an adjacent cage. Nasal washes were collected every 2 days from all animals beginning 2 days p.i. (1 day p.e.) for the detection of virus shedding. Each color bar represents the virus titer from an individual animal. The horizontal dashed red lines indicate the lower limit of detection.

The H4 AIVs in the present study can replicate in mice and have acquired the ability to bind to α-2,6-linked SAs. Therefore, we asked whether these H4 AIVs could transmit among mammals. First, we tested the 10 H4 viruses that bound to α-2,6-linked SAs in the solid-phase binding assay for their replicative ability in guinea pigs. Two animals were inoculated i.n. with 10^6.0^ EID_50_ of virus and euthanized 3 days p.i. The nasal wash and lung then were collected from each animal for virus titration in eggs. We found that viruses were detected in the nasal washes and lungs of guinea pigs inoculated with all 10 of these different H4 viruses. The virus titers were 0.8 to 4.0 log_10_ EID_50_/ml in nasal washes and 1.5 to 4.3 log_10_ EID_50_/ml in lungs (Table 3). Our data demonstrate that the H4 viruses can replicate as well in guinea pigs as in mice. We next investigated whether these viruses could transmit among guinea pigs by direct contact. Three guinea pigs were inoculated i.n. with 10^6.0^ EID_50_ of the test virus and housed in a cage within an isolator. Twenty-four hours later, three naive guinea pigs were placed in the same cage. Evidence of transmission was based on the detection of virus in the nasal wash and on seroconversion at the end of the 3-week observation period. As shown in Fig. 4, in the A/duck/Jiangxi/S3261/09 (DK/Jiangxi/S3261/09, H4N2)-, A/duck/Guangxi/S1211/10 (DK/Guangxi/S1211/10, H4N6)-, and A/duck/Guangdong/S4040/11 (DK/Guangdong/S4040/11, H4N6)-inoculated groups, virus was detected in the nasal washes of all three inoculated guinea pigs but not in any of the contact guinea pigs (Fig. 4B to D). In the A/duck/Hunan/S1012/09 (DK/Hunan/S1012/09, H4N6)-inoculated group, virus was detected in the nasal washes of all three inoculated guinea pigs and also was detected in the nasal wash of one of the three contact animals (Fig. 4A). In the A/duck/Fujian/S2169/12 (DK/Fujian/S2169/12, H4N6)-inoculated group, virus was detected in all three inoculated animals and two of the three contact animals (Fig. 4G). In the A/duck/Zhejiang/S2088/11 (DK/Zhejiang/S2088/11, H4N6)-, DK/Chongqing/S2086/12 (H4N8)-, A/goose/Guangdong/S1780/12 (GS/Guangdong/S1780/12, H4N2)-, DK/Jiangxi/S21055/12 (H4N2)-, and CK/Shandong/S2510/12 (H4N2)-inoculated groups, virus was detected in the nasal washes of all three inoculated guinea pigs and in all three contact guinea pigs (Fig. 4E, F, and H to J). Seroconversion was detected in all inoculated animals and in the contact animals that were virus positive in the nasal washes. One contact guinea pig in the DK/Fujian/S2169/12 (H4N6) group that was negative for virus detection also seroconverted (Table 3). These results indicate that the H4 viruses transmit among guinea pigs with various abilities, and some strains can transmit efficiently by direct contact in this mammalian host. Therefore, we selected 4 viruses, CK/Shandong/S2510/12 (H4N2), DK/Jiangxi/S21055/12 (H4N2), DK/Zhejiang/S2088/11 (H4N6), and DK/Chongqing/S2086/12 (H4N8), that transmitted to 100% of the direct-contact guinea pigs to evaluate their respiratory droplet transmission in this model. Five guinea pigs were inoculated i.n. with 10^6.0^ EID_50_ of the test virus and then housed individually in solid stainless steel cages within an isolator. Twenty-four hours later, five naive guinea pigs were each placed in a cage adjacent to the cage of an inoculated animal. Each pair of animals (one inoculated and one exposed) was separated by a double-layered net divider (4 cm apart) as described previously (13). Nasal washes were collected every 2 days from all of the animals beginning 2 days p.i. (1 day p.e.) for the detection of virus shedding. Sera were collected from all animals on day 21 p.i. for HI antibody detection. Respiratory droplet transmission was confirmed when virus was detected in the nasal washes and by seroconversion of the exposed animals at the end of the 3-week observation period. As shown in Fig. 5, virus was detected in all of the directly infected guinea pigs (Fig. 5A to D). In the DK/Jiangxi/S21055/12 (H4N2)-inoculated group, virus was not detected in any of the exposed guinea pigs (Fig. 5D). In the DK/Zhejiang/S2088/11 (H4N6)- and DK/Chongqing/S2086/12 (H4N8)-inoculated groups, each virus was detected in one of the five exposed guinea pigs (Fig. 5A and C). In the CK/Shandong/S2510/12 (H4N2)-inoculated group, virus was detected in two of the five exposed guinea pigs (Fig. 5B). Seroconversion was detected in the inoculated animals and in all exposed animals that were virus positive in nasal washes (Table 3). These results indicate that three of the four H4 AIVs tested can transmit between guinea pigs by respiratory droplet, albeit with limited efficiency. To investigate whether the respiratory droplet transmissibility of the H4 AIVs in this study was conferred by mutations acquired during virus replication in guinea pigs, we amplified the eight viral gene segments from nasal wash samples that were recovered from guinea pigs exposed to DK/Zhejiang/S2088/11 (H4N6), CK/Shandong/S2510/12 (H4N2), or DK/Chongqing/S2086/12 (H4N8) on day 7 p.e. and that were positive for virus isolation (a total of 4 samples). The amplified PCR products were inserted into the pEASY-Blunt Zero cloning vector. We sequenced 15 positive clones of each construct to examine the potential mutations in the viral genome. Sequencing analysis revealed no mutations in any of the viruses. These results suggest that the transmissibility observed in this study is an intrinsic property of H4 AIVs.

**TABLE 3:** Transmission of H4 avian influenza viruses in guinea pigs

Virus	Genotype	Virus titer^*a*^ (log_10_ EID_50_/ml)		Direct-contact transmission			Respiratory droplet transmission		
				Seroconversion^*b*^ (HI titers), no. positive/total no.		Transmission efficiency	Seroconversion^*b*^ (HI titers), no. positive/total no.		Transmission efficiency
		Nasal wash	Lung	Inoculated	Exposed		Inoculated	Exposed	
DK/Jiangxi/S3261/09 (H4N2)	A1	2.5, 1.8	3.5, 2.8	3/3 (20–40)	0/3	None	ND	ND	ND
DK/Guangdong/S4040/11 (H4N2)	A3	1.0, 2.8	2.8, 2.8	3/3 (20–160)	0/3	None	ND	ND	ND
GS/Guangdong/S1780/12 (H4N2)	A6	1.5, 1.8	2.3, 3.3	3/3 (80–160)	3/3 (20–80)	Highly efficient	ND	ND	ND
DK/Jiangxi/S21055/12 (H4N2)	A10	3.8, 3.3	3.0, 3.8	3/3 (160)	3/3 (40–160)	Highly efficient	5/5 (40–80)	0/5	None
CK/Shandong/S2510/12 (H4N2)	A11	2.8, 0.8	1.8, 2.8	3/3 (20–80)	3/3 (20–40)	Highly efficient	5/5 (160–320)	2/5 (160–320)	Less efficient
DK/Hunan/S1012/09 (H4N6)	C2	2.8, 2.8	2.0, 2.3	3/3 (40–80)	1/3 (40)	Less efficient	ND	ND	ND
DK/Guangxi/S1211/10 (H4N6)	C5	1.5, 3.3	1.5, 2.5	3/3 (20–40)	0/3	None	ND	ND	ND
DK/Zhejiang/S2088/11 (H4N6)	C10	3.3, 2.8	3.8, 2.3	3/3 (80–160)	3/3 (40–320)	Highly efficient	5/5 (160–640)	1/5 (160)	Less efficient
DK/Fujian/S2169/12 (H4N6)	C13	2.5, 3.5	1.5, 3.3	3/3 (40–80)	3/3 (20–40)	Highly efficient	ND	ND	ND
DK/Chongqing/S2086/12 (H4N8)	D3	2.8, 4.0	4.3, 2.8	3/3 (80–160)	3/3 (40–80)	Highly efficient	5/5 (160–320)	1/5 (160)	Less efficient

a Viral titers are shown as individual titers of the two virus-inoculated guinea pigs.

b Sera were collected from the guinea pigs 3 weeks after virus inoculation or exposure; these animals were used for the transmission studies shown in Fig. 4 and 5. HI, hemagglutinin inhibition; ND, not done; None, virus was not transmitted from the inoculated animals to the direct-contact or exposed animals.

## DISCUSSION

Due to the zoonotic nature of AIVs, global attention has been paid to their prevention and control. The outbreak of H7N9 human infection in 2013 in China taught us that low-pathogenicity AIVs can lead to severe infection and even death in humans. Therefore, attention also must be paid to the potential threat to humans posed by low-pathogenicity AIVs. In this study, we systematically characterized H4 AIVs isolated from live poultry markets in 14 provinces of China between 2009 and 2012. Our phylogenetic analysis revealed that the H4 AIVs circulating in nature have various genetic constellations, with at least 32 genotypes, indicating that complex and extensive reassortment events have occurred among the H4 AIVs circulating in live poultry markets. We used BALB/c mice as a model to evaluate the replication and virulence of H4 AIVs and found that these viruses could replicate in mice without prior adaptation. Our analysis of receptor binding specificity revealed that H4 AIVs can bind to human-type receptors. Our transmission studies demonstrated that some of the H4 viruses can transmit between guinea pigs with 100% efficiency by direct contact, and that three out of four viruses tested transmitted via respiratory droplet, albeit with limited efficiency.

Influenza virus initiates infection by binding to the sialic acid receptors on the surface of polarized host cells. In 1983, Rogers and Paulson first demonstrated that H3 avian viruses preferentially bound to erythrocytes containing SAs with the α-2,3 linkage, whereas human isolates preferentially bind to erythrocytes with α-2,6-linked SAs (70). Later studies with viruses of the H1 and H2 subtypes suggested that this receptor binding pattern is a general property of influenza viruses (71, 72). However, this binding pattern does not appear to be absolute, given that many recent avian virus isolates of different subtypes have binding affinity for α-2,6-linked SAs (20, 25, 29, 31, 57, 73). In the present study, all of the H4 viruses tested could at least partially bind to α-2,6-linked SAs. We also demonstrated that the earliest H4 isolate, DK/Czechoslovakia/56, showed this receptor binding preference. These results indicate that the H4 virus is distinct in its receptor binding property, showing intrinsic binding to the human-type receptors. It is well accepted that the transition from avian-type to human-type receptor binding preference is a crucial step for influenza viruses to replicate efficiently and transmit in humans (74). However, the specific amino acids that determine receptor binding specificity vary among the different HA subtypes. For H1 viruses, the substitutions of E190D and G225D are critical for the shift from α-2,3- to α-2,6-linked receptor recognition (75, 76). For H2 and H3 viruses, the substitutions Q226L and G228S in HA can confer a complete switch from α-2,3- to α-2,6-linked receptor binding specificity (71, 77, 78). Extensive studies have revealed that a number of mutations can allow the H5N1 viruses to bind partially to human-type receptors; these mutations include S125N, L133V/A138V, 133 deletion/I155T, G143R, S159N, T160A, N186K, D187G, K193R, Q196H, Q196R, N197K, V214I, Q226L, S227N, G228S, and S239P (14, 57, 61, 67–69, 79, 80). However, viruses with these mutations retain their affinity for avian-type receptors. Recently, several studies with viruses generated by use of reverse genetics have shown that specific combinations of mutations in HA, such as Q196R/Q226L/G228S, N224K/Q226L, or Q226L/G228S, result in the switch from α-2,3- to α-2,6-linked receptor binding preference (11, 12, 14). The recently emerged H7N9 virus exhibits substantial binding affinity for α-2,6-linked glycans, which is conferred by the Q226L, I243V, and G186V mutations in its HA (20, 81, 82). The H9N2 AIVs have been widely circulating around the world, and the Q226L mutation has been increasingly found in recent H9N2 isolates (25, 83). This mutation is reported to contribute to the human-type receptor binding specificity of H9N2 virus (84). Our recent study demonstrated that the majority of contemporary H9N2 viruses from poultry have acquired the ability to preferentially bind to human-type receptors, similar to human influenza viruses, a property shown to be conferred by the Q226L and I155T mutations in HA (25). It should be noted that the change in receptor binding specificity that occurs during adaptation from avian to human hosts is thought to include not only the acquisition of binding to the α-2,6-linked SAs but also the loss of binding to the α-2,3-linked SAs. The 36 H4 viruses in this study, as well as the prototype avian H4 virus DK/Czechoslovakia/56, have identical receptor binding sites. We found that all of the H4 AIVs tested retained high affinity for α-2,3-linked glycans even though they could bind to α-2,6-linked glycans. Mutagenesis studies indicated that multiple amino acids at or around the HA receptor binding sites were important to the human-type receptor binding specificity of these H4 viruses and that the role played by the asparagine residue at position 193 was essential. Bateman et al. previously reported that the first swine H4N6 isolate from Canada in 1999, which possessed 226L and 228S in its HA, had higher affinity for α-2,6-linked SAs and higher infectivity in primary swine and human respiratory epithelial cells than the mutant virus containing 226Q and 228G in its HA (85). Their findings suggest that the H4 viruses are predisposed to acquire the Q226L/G228S mutations during their circulation. Therefore, more attention should be paid to such mutant viruses during the H4 AIV surveillance, especially in mammalian hosts, such as pigs.

Influenza viruses evolve over time by two mechanisms: mutation and reassortment. Compared with mutation, the reassortment process can result in significant phenotype changes, because a set of eight segments from viruses of different origins can be gathered to create the reassortant viruses. Although the gene segments of different viruses are not freely exchanged during the reassortment process because of incompatibility at the nucleotide or protein level (86, 87), reassortment is an effective means of generating human pandemic viruses, as exemplified by three of the four human pandemics since the beginning of the last century being caused by reassortant viruses (4, 5, 7). In the present study, we demonstrated that the H4 AIVs in live poultry markets are undergoing complex and frequent reassortment events. The extensive reassortment of H4 AIVs is worrisome because it may produce hybrid viruses that can jump to humans and cause major public health issues, as occurred with the newly emerged H7N9 influenza virus, which is a triple reassortant, acquiring its HA, NA, and six internal genes from different ancestors (15, 88). The intrinsic difficulty of surveillance of low-pathogenicity AIVs is that they do not cause symptoms in infected poultry, allowing the viruses to spread silently. To address this difficulty, intensive surveillance must be conducted if newly emerged reassortant viruses are to be detected early. Other control measures also should be strengthened; for example, the closure of live poultry markets should be seriously considered to prevent humans from being infected by the viruses in poultry.

The concern caused by low-pathogenicity AIVs has increased in recent years. During their replication in mammals, including humans, the low-pathogenicity AIVs may acquire new mutations and become more virulent or more transmissible. Song et al. previously serially passaged an avirulent wild bird H5N2 virus in the lungs of mice and identified a PA T97I mutation that enhanced virus virulence in mice (89). For the recently emerged H7N9 viruses, all virus isolates from poultry possessed 627E and 701D in their PB2 protein, whereas most of the human isolates acquired 627K or 701N (20, 90, 91), the two well-documented virulence signatures of AIVs in mammalian hosts. During the passage of H9N2 viruses in mice, several residues were reported to increase the pathogenicity of the mutant viruses, including PB2 M147L, F404L, and E627K (92–94). In our recent study, we found that the E627K or D701N mutation easily emerged in both inoculated and exposed ferrets, and it significantly increased the virulence and enhanced the transmissibility of H9N2 viruses in ferrets (25). It has been shown that H4 AIVs can replicate well in mammals, including mice and pigs (32, 35, 95). Serological studies demonstrated that poultry farmers and workers have been infected with various subtypes of AIVs, including H4, in the United States and Lebanon (54, 55). In the present study, all 32 H4 viruses tested could replicate in mice; the 10 viruses examined also could replicate in guinea pigs. Our results clearly demonstrate the potential threat posed by the H4 AIVs to public health. Therefore, the possible infection of humans or other mammalian hosts by H4 AIVs in the field, and the appearance of virulence markers in the virus genome, such as PB2 E627K or D701N, must be closely monitored.

The acquisition of efficient respiratory droplet transmissibility among humans is a prerequisite for the emergence of an influenza pandemic. In the past decade, great efforts have been made to investigate the pandemic potential of various subtypes of AIVs. Ferrets and guinea pigs are widely used in influenza virus transmission research, because these two animal models are readily susceptible to infection with human influenza viruses, demonstrating robust viral replication and efficient virus transmission to exposed animals. These studies have demonstrated that experimentally generated H5N1 and H9N2 viruses can acquire the ability to transmit between ferrets or guinea pigs via respiratory droplet after gaining specific mutations or by reassorting with human influenza viruses (11–14, 24, 96). We and others also have shown that certain natural strains of H7N9 and H9N2 viruses can transmit among ferrets by respiratory droplet (16–20, 25). In addition, Karlsson et al. recently reported that an avian H3N8 virus isolated from harbor seals can transmit among ferrets by respiratory droplet (97). Here, we demonstrated that 7 of 10 H4 viruses tested could be transmitted to animals via direct contact, and, more strikingly, three of four of these viruses could be transmitted to exposed animals via respiratory droplet. Influenza virus transmission mediated by respiratory droplet can be further divided into droplet spay transmission, in which the respiratory droplet released by the infected human or animal directly impacts the respiratory mucosa of susceptible recipients, and aerosol transmission, which is caused by a small, light aerosol (also called droplet nuclei) that can suspend in the air for minutes or hours (98). We demonstrated that three of four H4 viruses acquired inefficient respiratory droplet transmissibility. However, it remains unknown whether these H4 viruses are capable of inefficient aerosol transmission by small-nucleus droplet. Given that the H4 viruses have acquired the ability to replicate in mammalian hosts, it is possible that they will eventually evolve into viruses with efficient respiratory droplet transmissibility through the accumulation of mutations or reassortment, necessitating frequent risk analysis.

## Supplementary Material

Supplemental material
