# Supplementary material for: Genetics, Receptor Binding, Replication, and Mammalian Transmission of H4 Avian Influenza Viruses Isolated from Live Poultry Markets in China
Source: J Virol. 2016 Jan 15;90(3):1455–69. doi: 10.1128/JVI.02692-15 (PMC4719592; doi:10.1128/JVI.02692-15)
Supplement: Supplemental material [file supp_90_3_1455__index.html]

Genetics, Receptor Binding, Replication, and Mammalian Transmission of H4 Avian Influenza Viruses Isolated from Live Poultry Markets in China — Supplemental material 

# Genetics, Receptor Binding, Replication, and Mammalian Transmission of H4 Avian Influenza Viruses Isolated from Live Poultry Markets in China

## Supplemental material

- Supplemental file 1 -

  Fig. S1 (Phylogenetic analyses of the six internal genes of H4 avian influenza viruses isolated from live poultry markets in China between 2009 and 2012.)

  PDF, 181K
