# Supplementary material for: Genetics, Receptor Binding, Replication, and Mammalian Transmission of H4 Avian Influenza Viruses Isolated from Live Poultry Markets in China
Source: J Virol. 2016 Jan 15;90(3):1455–69. doi: 10.1128/JVI.02692-15 (PMC4719592; doi:10.1128/JVI.02692-15)
Supplement: Supplemental material [file JVI.02692-15_zjv999091247so1.pdf]

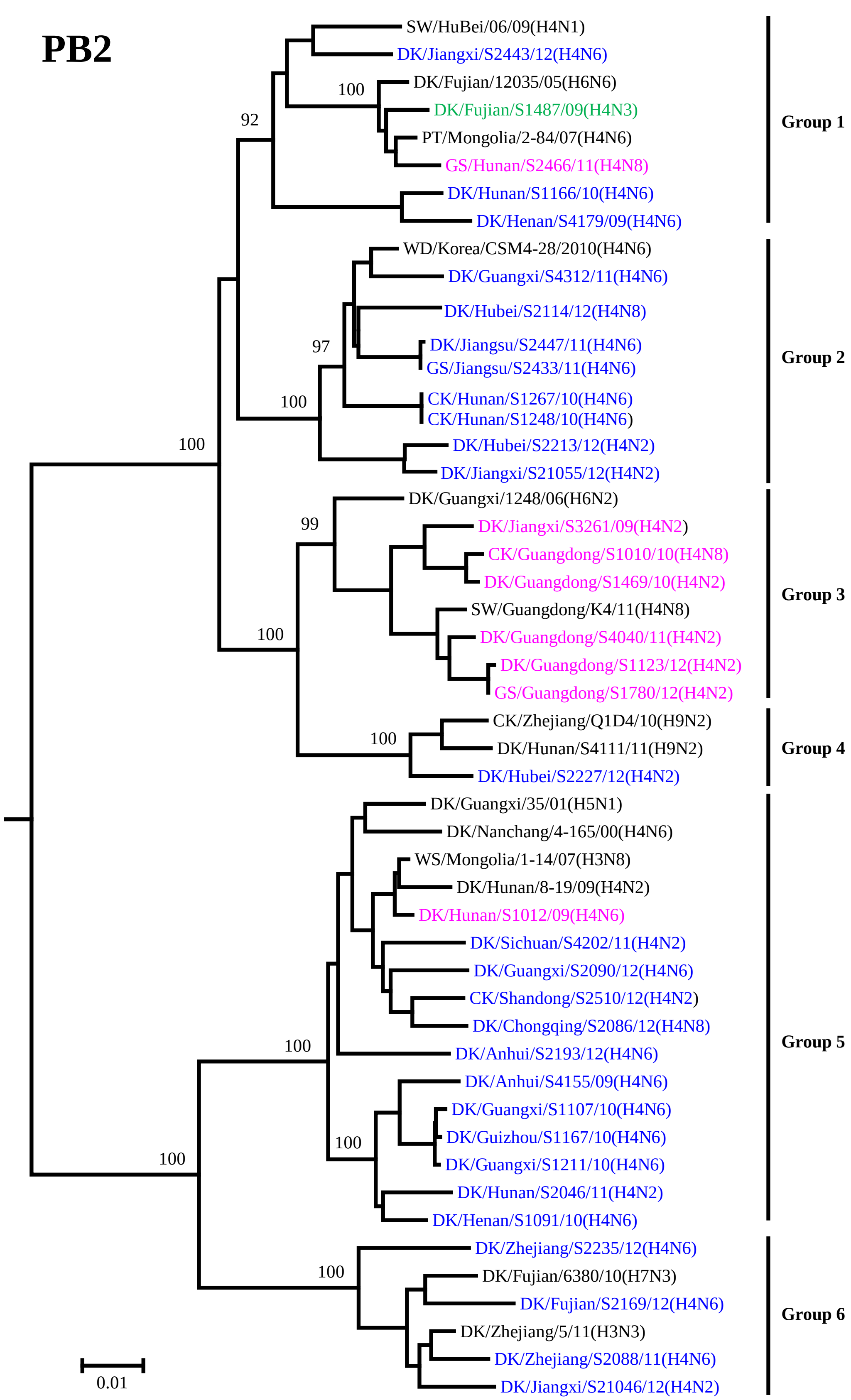
PB2
SW/HuBei/06/09(H4N1)
DK/Jiangxi/S2443/12(H4N6)
DK/Fujian/12035/05(H6N6)
DK/Fujian/S1487/09(H4N3)
PT/Mongolia/2-84/07(H4N6)
GS/Hunan/S2466/11(H4N8)
DK/Hunan/S1166/10(H4N6)
DK/Henan/S4179/09(H4N6)
WD/Korea/CSM4-28/2010(H4N6)
DK/Guangxi/S4312/11(H4N6)
DK/Hubei/S2114/12(H4N8)
DK/Jiangsu/S2447/11(H4N6)
GS/Jiangsu/S2433/11(H4N6)
CK/Hunan/S1267/10(H4N6)
CK/Hunan/S1248/10(H4N6)
DK/Hubei/S2213/12(H4N2)
DK/Jiangxi/S21055/12(H4N2)
DK/Guangxi/1248/06(H6N2)
DK/Jiangxi/S3261/09(H4N2)
CK/Guangdong/S1010/10(H4N8)
DK/Guangdong/S1469/10(H4N2)
SW/Guangdong/K4/11(H4N8)
DK/Guangdong/S4040/11(H4N2)
DK/Guangdong/S1123/12(H4N2)
GS/Guangdong/S1780/12(H4N2)
CK/Zhejiang/Q1D4/10(H9N2)
DK/Hunan/S4111/11(H9N2)
DK/Hubei/S2227/12(H4N2)
DK/Guangxi/35/01(H5N1)
DK/Nanchang/4-165/00(H4N6)
WS/Mongolia/1-14/07(H3N8)
DK/Hunan/8-19/09(H4N2)
DK/Hunan/S1012/09(H4N6)
DK/Sichuan/S4202/11(H4N2)
DK/Guangxi/S2090/12(H4N6)
CK/Shandong/S2510/12(H4N2)
DK/Chongqing/S2086/12(H4N8)
DK/Anhui/S2193/12(H4N6)
DK/Anhui/S4155/09(H4N6)
DK/Guangxi/S1107/10(H4N6)
DK/Guizhou/S1167/10(H4N6)
DK/Guangxi/S1211/10(H4N6)
DK/Hunan/S2046/11(H4N2)
DK/Henan/S1091/10(H4N6)
DK/Zhejiang/S2235/12(H4N6)
DK/Fujian/6380/10(H7N3)
DK/Fujian/S2169/12(H4N6)
DK/Zhejiang/5/11(H3N3)
DK/Zhejiang/S2088/11(H4N6)
DK/Jiangxi/S21046/12(H4N2)
Group 1
Group 2
Group 3
Group 4
Group 5
Group 6
100
92
97
100
100
99
100
100
100
100
100
100
0.01

Fig. S1A

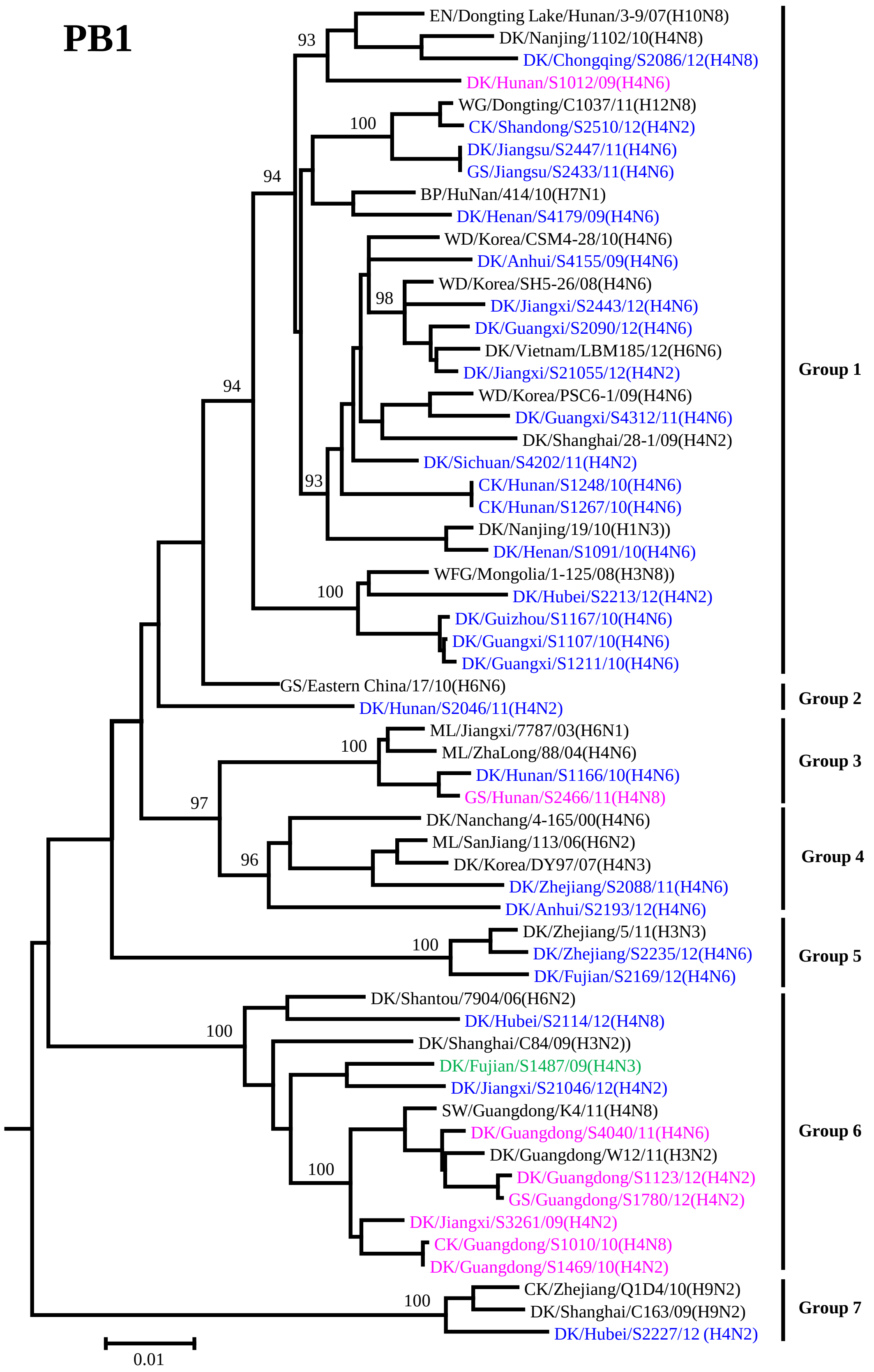
PB1
EN/Dongting Lake/Hunan/3-9/07(H10N8)
DK/Nanjing/1102/10(H4N8)
DK/Chongqing/S2086/12(H4N8)
DK/Hunan/S1012/09(H4N6)
WG/Dongting/C1037/11(H12N8)
CK/Shandong/S2510/12(H4N2)
DK/Jiangsu/S2447/11(H4N6)
GS/Jiangsu/S2433/11(H4N6)
BP/HuNan/414/10(H7N1)
DK/Henan/S4179/09(H4N6)
WD/Korea/CSM4-28/10(H4N6)
DK/Anhui/S4155/09(H4N6)
WD/Korea/SH5-26/08(H4N6)
DK/Jiangxi/S2443/12(H4N6)
DK/Guangxi/S2090/12(H4N6)
DK/Vietnam/LBM185/12(H6N6)
DK/Jiangxi/S21055/12(H4N2)
WD/Korea/PSC6-1/09(H4N6)
DK/Guangxi/S4312/11(H4N6)
DK/Shanghai/28-1/09(H4N2)
DK/Sichuan/S4202/11(H4N2)
CK/Hunan/S1248/10(H4N6)
CK/Hunan/S1267/10(H4N6)
DK/Nanjing/19/10(H1N3))
DK/Henan/S1091/10(H4N6)
WFG/Mongolia/1-125/08(H3N8))
DK/Hubei/S2213/12(H4N2)
DK/Guizhou/S1167/10(H4N6)
DK/Guangxi/S1107/10(H4N6)
DK/Guangxi/S1211/10(H4N6)
GS/Eastern China/17/10(H6N6)
DK/Hunan/S2046/11(H4N2)
ML/Jiangxi/7787/03(H6N1)
ML/ZhaLong/88/04(H4N6)
DK/Hunan/S1166/10(H4N6)
GS/Hunan/S2466/11(H4N8)
DK/Nanchang/4-165/00(H4N6)
ML/SanJiang/113/06(H6N2)
DK/Korea/DY97/07(H4N3)
DK/Zhejiang/S2088/11(H4N6)
DK/Anhui/S2193/12(H4N6)
DK/Zhejiang/5/11(H3N3)
DK/Zhejiang/S2235/12(H4N6)
DK/Fujian/S2169/12(H4N6)
DK/Shantou/7904/06(H6N2)
DK/Hubei/S2114/12(H4N8)
DK/Shanghai/C84/09(H3N2))
DK/Fujian/S1487/09(H4N3)
DK/Jiangxi/S21046/12(H4N2)
SW/Guangdong/K4/11(H4N8)
DK/Guangdong/S4040/11(H4N6)
DK/Guangdong/W12/11(H3N2)
DK/Guangdong/S1123/12(H4N2)
GS/Guangdong/S1780/12(H4N2)
DK/Jiangxi/S3261/09(H4N2)
CK/Guangdong/S1010/10(H4N8)
DK/Guangdong/S1469/10(H4N2)
CK/Zhejiang/Q1D4/10(H9N2)
DK/Shanghai/C163/09(H9N2)
DK/Hubei/S2227/12 (H4N2)
93
100
94
98
94
93
100
100
97
96
100
100
100
100
Group 1
Group 2
Group 3
Group 4
Group 5
Group 6
Group 7
0.01

Fig. S1B

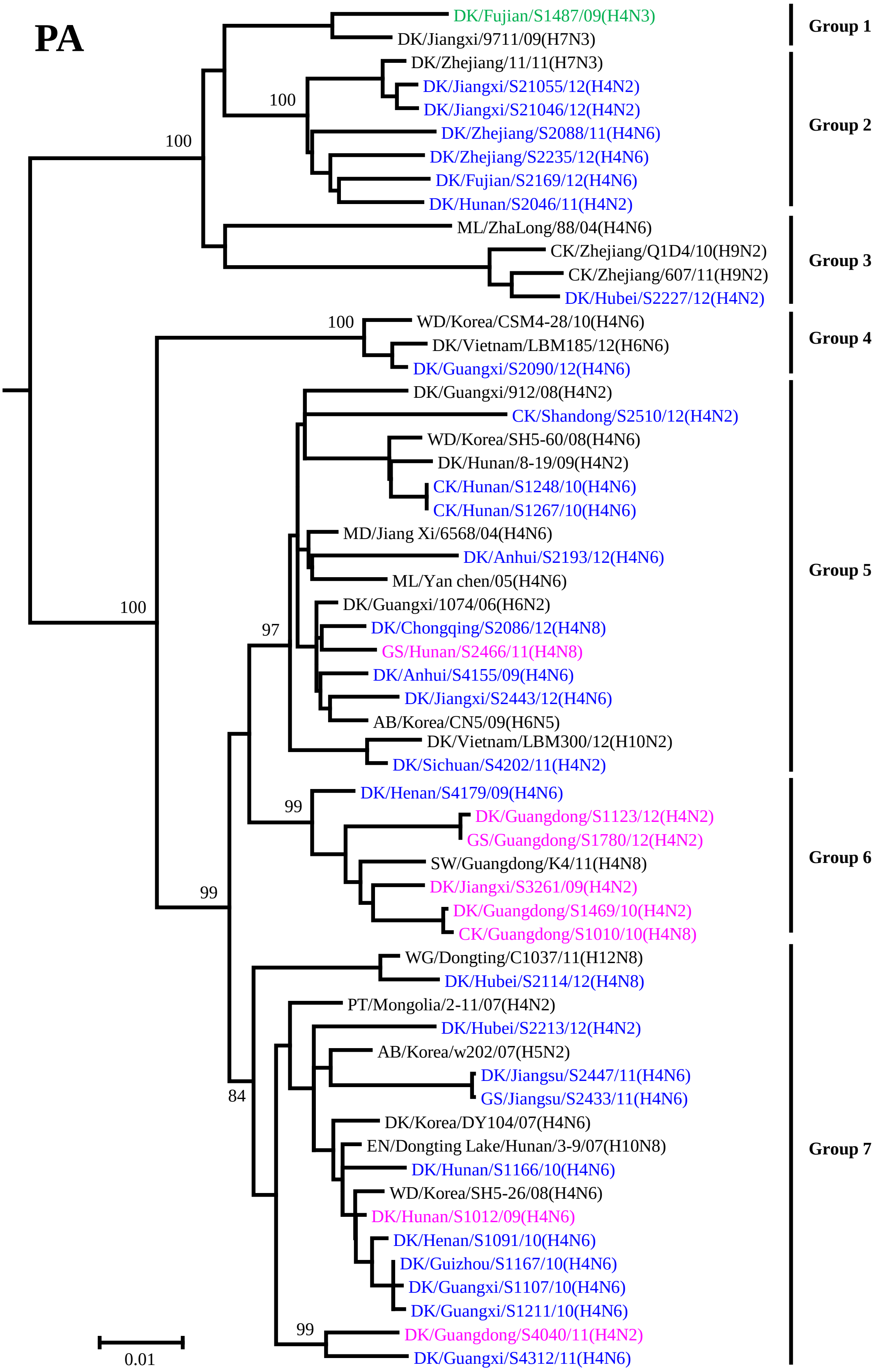

PA
DK/Fujian/S1487/09(H4N3)
DK/Jiangxi/9711/09(H7N3)
DK/Zhejiang/11/11(H7N3)
DK/Jiangxi/S21055/12(H4N2)
DK/Jiangxi/S21046/12(H4N2)
DK/Zhejiang/S2088/11(H4N6)
DK/Zhejiang/S2235/12(H4N6)
DK/Fujian/S2169/12(H4N6)
DK/Hunan/S2046/11(H4N2)
ML/ZhaLong/88/04(H4N6)
CK/Zhejiang/Q1D4/10(H9N2)
CK/Zhejiang/607/11(H9N2)
DK/Hubei/S2227/12(H4N2)
WD/Korea/CSM4-28/10(H4N6)
DK/Vietnam/LBM185/12(H6N6)
DK/Guangxi/S2090/12(H4N6)
DK/Guangxi/912/08(H4N2)
CK/Shandong/S2510/12(H4N2)
WD/Korea/SH5-60/08(H4N6)
DK/Hunan/8-19/09(H4N2)
CK/Hunan/S1248/10(H4N6)
CK/Hunan/S1267/10(H4N6)
MD/Jiang Xi/6568/04(H4N6)
DK/Anhui/S2193/12(H4N6)
ML/Yan chen/05(H4N6)
DK/Guangxi/1074/06(H6N2)
DK/Chongqing/S2086/12(H4N8)
GS/Hunan/S2466/11(H4N8)
DK/Anhui/S4155/09(H4N6)
DK/Jiangxi/S2443/12(H4N6)
AB/Korea/CN5/09(H6N5)
DK/Vietnam/LBM300/12(H10N2)
DK/Sichuan/S4202/11(H4N2)
DK/Henan/S4179/09(H4N6)
DK/Guangdong/S1123/12(H4N2)
GS/Guangdong/S1780/12(H4N2)
SW/Guangdong/K4/11(H4N8)
DK/Jiangxi/S3261/09(H4N2)
DK/Guangdong/S1469/10(H4N2)
CK/Guangdong/S1010/10(H4N8)
WG/Dongting/C1037/11(H12N8)
DK/Hubei/S2114/12(H4N8)
PT/Mongolia/2-11/07(H4N2)
DK/Hubei/S2213/12(H4N2)
AB/Korea/w202/07(H5N2)
DK/Jiangsu/S2447/11(H4N6)
GS/Jiangsu/S2433/11(H4N6)
DK/Korea/DY104/07(H4N6)
EN/Dongting Lake/Hunan/3-9/07(H10N8)
DK/Hunan/S1166/10(H4N6)
WD/Korea/SH5-26/08(H4N6)
DK/Hunan/S1012/09(H4N6)
DK/Henan/S1091/10(H4N6)
DK/Guizhou/S1167/10(H4N6)
DK/Guangxi/S1107/10(H4N6)
DK/Guangxi/S1211/10(H4N6)
DK/Guangdong/S4040/11(H4N2)
DK/Guangxi/S4312/11(H4N6)
100
100
100
100
97
99
99
84
99
0.01
Group 1
Group 2
Group 3
Group 4
Group 5
Group 6
Group 7

Fig. S1C

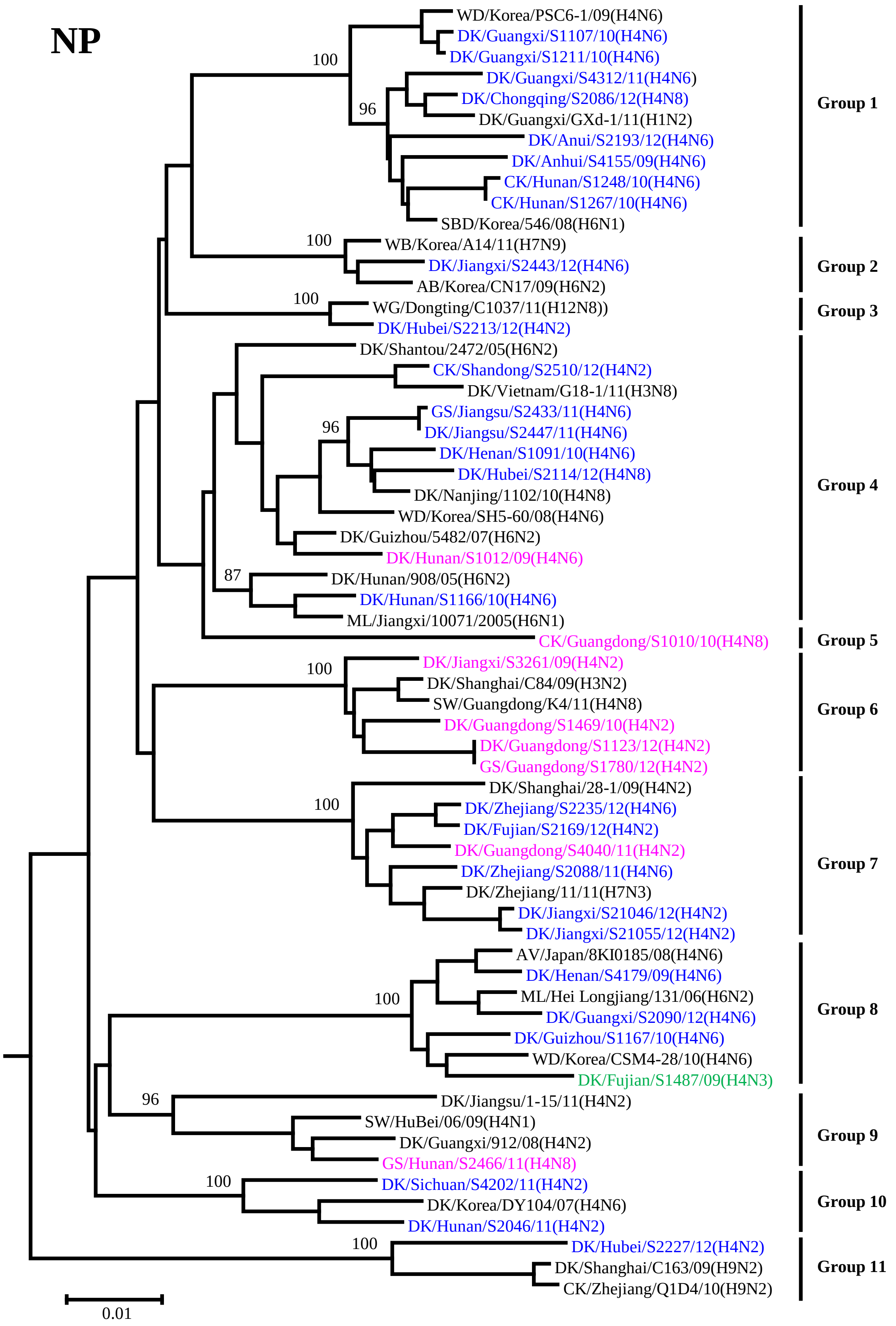
NP
WD/Korea/PSC6-1/09(H4N6)
DK/Guangxi/S1107/10(H4N6)
DK/Guangxi/S1211/10(H4N6)
DK/Guangxi/S4312/11(H4N6)
DK/Chongqing/S2086/12(H4N8)
DK/Guangxi/GXd-1/11(H1N2)
DK/Anui/S2193/12(H4N6)
DK/Anhui/S4155/09(H4N6)
CK/Hunan/S1248/10(H4N6)
CK/Hunan/S1267/10(H4N6)
SBD/Korea/546/08(H6N1)
WB/Korea/A14/11(H7N9)
DK/Jiangxi/S2443/12(H4N6)
AB/Korea/CN17/09(H6N2)
WG/Dongting/C1037/11(H12N8))
DK/Hubei/S2213/12(H4N2)
DK/Shantou/2472/05(H6N2)
CK/Shandong/S2510/12(H4N2)
DK/Vietnam/G18-1/11(H3N8)
GS/Jiangsu/S2433/11(H4N6)
DK/Jiangsu/S2447/11(H4N6)
DK/Henan/S1091/10(H4N6)
DK/Hubei/S2114/12(H4N8)
DK/Nanjing/1102/10(H4N8)
WD/Korea/SH5-60/08(H4N6)
DK/Guizhou/5482/07(H6N2)
DK/Hunan/S1012/09(H4N6)
DK/Hunan/908/05(H6N2)
DK/Hunan/S1166/10(H4N6)
ML/Jiangxi/10071/2005(H6N1)
CK/Guangdong/S1010/10(H4N8)
DK/Jiangxi/S3261/09(H4N2)
DK/Shanghai/C84/09(H3N2)
SW/Guangdong/K4/11(H4N8)
DK/Guangdong/S1469/10(H4N2)
DK/Guangdong/S1123/12(H4N2)
GS/Guangdong/S1780/12(H4N2)
DK/Shanghai/28-1/09(H4N2)
DK/Zhejiang/S2235/12(H4N6)
DK/Fujian/S2169/12(H4N2)
DK/Guangdong/S4040/11(H4N2)
DK/Zhejiang/S2088/11(H4N6)
DK/Zhejiang/11/11(H7N3)
DK/Jiangxi/S21046/12(H4N2)
DK/Jiangxi/S21055/12(H4N2)
AV/Japan/8KI0185/08(H4N6)
DK/Henan/S4179/09(H4N6)
ML/Hei Longjiang/131/06(H6N2)
DK/Guangxi/S2090/12(H4N6)
DK/Guizhou/S1167/10(H4N6)
WD/Korea/CSM4-28/10(H4N6)
DK/Fujian/S1487/09(H4N3)
DK/Jiangsu/1-15/11(H4N2)
SW/HuBei/06/09(H4N1)
DK/Guangxi/912/08(H4N2)
GS/Hunan/S2466/11(H4N8)
DK/Sichuan/S4202/11(H4N2)
DK/Korea/DY104/07(H4N6)
DK/Hunan/S2046/11(H4N2)
DK/Hubei/S2227/12(H4N2)
DK/Shanghai/C163/09(H9N2)
CK/Zhejiang/Q1D4/10(H9N2)
100
96
100
100
96
87
100
100
100
96
100
100
Group 1
Group 2
Group 3
Group 4
Group 5
Group 6
Group 7
Group 8
Group 9
Group 10
Group 11
0.01

Fig. S1D

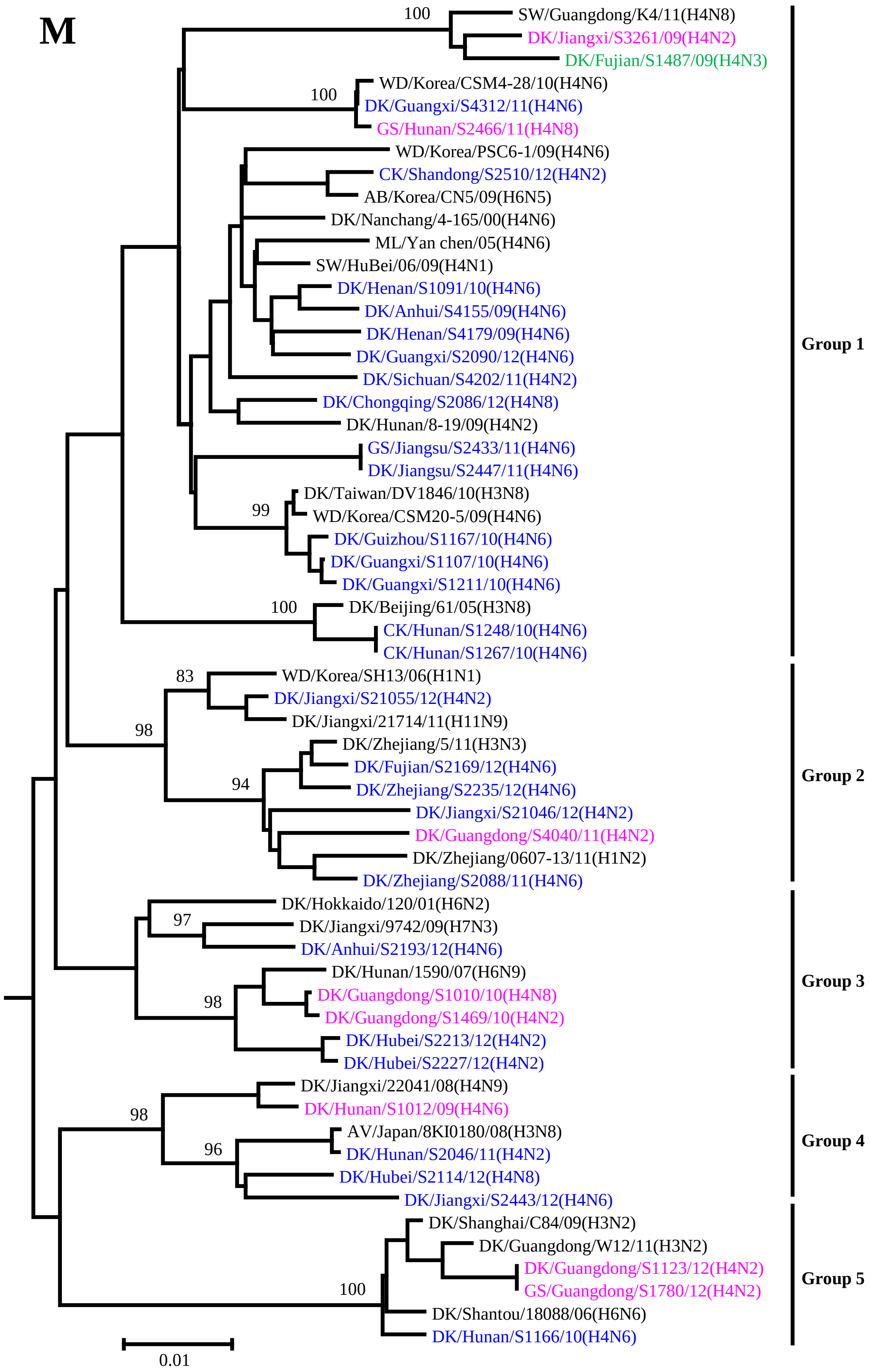
M
SW/Guangdong/K4/11(H4N8)
DK/Jiangxi/S3261/09(H4N2)
DK/Fujian/S1487/09(H4N3)
WD/Korea/CSM4-28/10(H4N6)
DK/Guangxi/S4312/11(H4N6)
GS/Hunan/S2466/11(H4N8)
WD/Korea/PSC6-1/09(H4N6)
CK/Shandong/S2510/12(H4N2)
AB/Korea/CN5/09(H6N5)
DK/Nanchang/4-165/00(H4N6)
ML/Yan chen/05(H4N6)
SW/HuBei/06/09(H4N1)
DK/Henan/S1091/10(H4N6)
DK/Anhui/S4155/09(H4N6)
DK/Henan/S4179/09(H4N6)
DK/Guangxi/S2090/12(H4N6)
DK/Sichuan/S4202/11(H4N2)
DK/Chongqing/S2086/12(H4N8)
DK/Hunan/8-19/09(H4N2)
GS/Jiangsu/S2433/11(H4N6)
DK/Jiangsu/S2447/11(H4N6)
DK/Taiwan/DV1846/10(H3N8)
WD/Korea/CSM20-5/09(H4N6)
DK/Guizhou/S1167/10(H4N6)
DK/Guangxi/S1107/10(H4N6)
DK/Guangxi/S1211/10(H4N6)
DK/Beijing/61/05(H3N8)
CK/Hunan/S1248/10(H4N6)
CK/Hunan/S1267/10(H4N6)
Group 1
WD/Korea/SH13/06(H1N1)
DK/Jiangxi/S21055/12(H4N2)
DK/Jiangxi/21714/11(H11N9)
DK/Zhejiang/5/11(H3N3)
DK/Fujian/S2169/12(H4N6)
DK/Zhejiang/S2235/12(H4N6)
DK/Jiangxi/S21046/12(H4N2)
DK/Guangdong/S4040/11(H4N2)
DK/Zhejiang/0607-13/11(H1N2)
DK/Zhejiang/S2088/11(H4N6)
Group 2
DK/Hokkaido/120/01(H6N2)
DK/Jiangxi/9742/09(H7N3)
DK/Anhui/S2193/12(H4N6)
DK/Hunan/1590/07(H6N9)
DK/Guangdong/S1010/10(H4N8)
DK/Guangdong/S1469/10(H4N2)
DK/Hubei/S2213/12(H4N2)
DK/Hubei/S2227/12(H4N2)
Group 3
DK/Jiangxi/22041/08(H4N9)
DK/Hunan/S1012/09(H4N6)
AV/Japan/8KI0180/08(H3N8)
DK/Hunan/S2046/11(H4N2)
DK/Hubei/S2114/12(H4N8)
DK/Jiangxi/S2443/12(H4N6)
Group 4
DK/Shanghai/C84/09(H3N2)
DK/Guangdong/W12/11(H3N2)
DK/Guangdong/S1123/12(H4N2)
GS/Guangdong/S1780/12(H4N2)
DK/Shantou/18088/06(H6N6)
DK/Hunan/S1166/10(H4N6)
Group 5
100
100
99
100
83
98
94
97
98
98
96
100
0.01

Fig. S1E

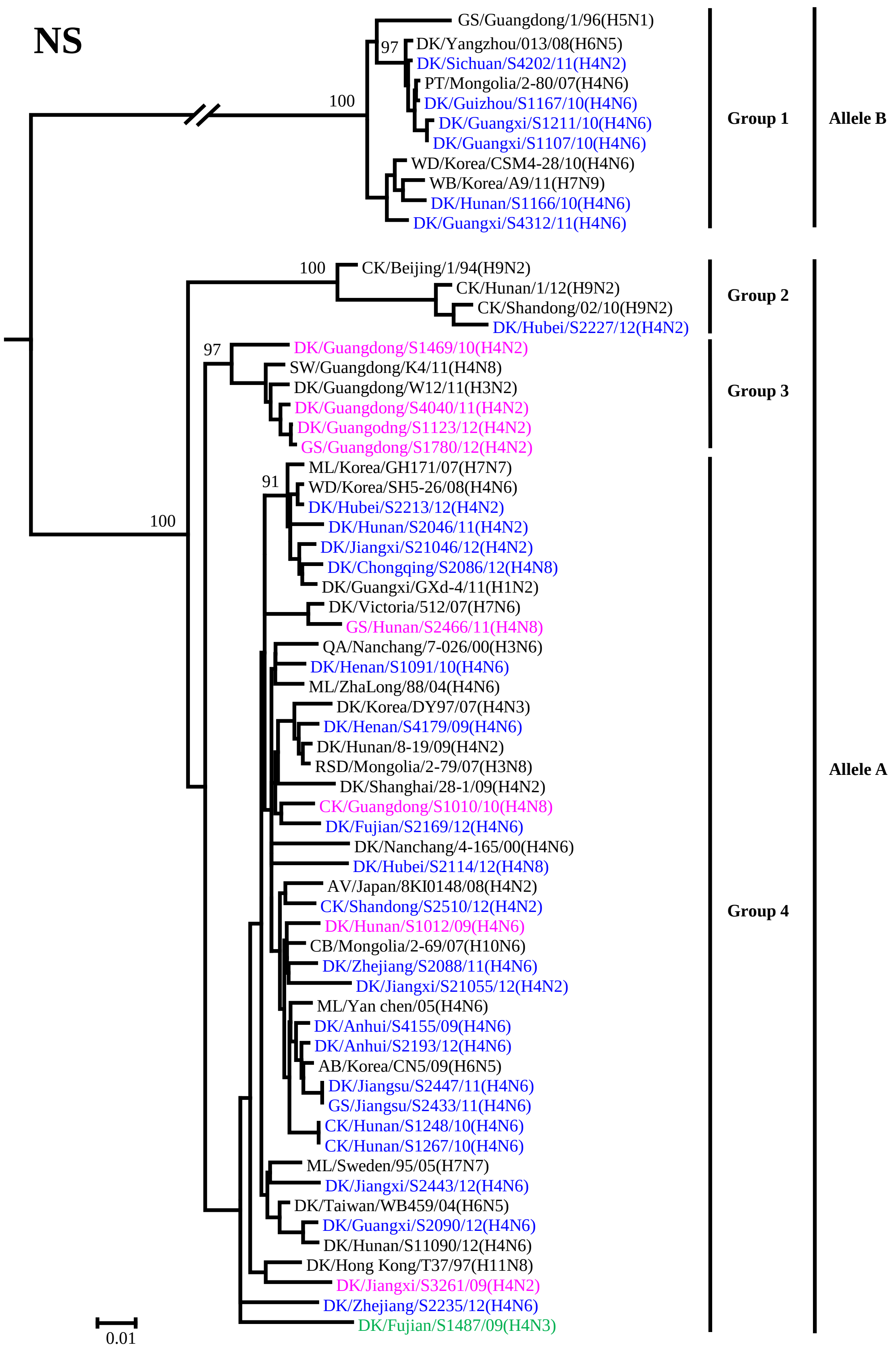
NS
GS/Guangdong/1/96(H5N1)
97
DK/Yangzhou/013/08(H6N5)
DK/Sichuan/S4202/11(H4N2)
PT/Mongolia/2-80/07(H4N6)
DK/Guizhou/S1167/10(H4N6)
100
DK/Guangxi/S1211/10(H4N6)
DK/Guangxi/S1107/10(H4N6)
WD/Korea/CSM4-28/10(H4N6)
WB/Korea/A9/11(H7N9)
DK/Hunan/S1166/10(H4N6)
DK/Guangxi/S4312/11(H4N6)
Group 1
Allele B
100
CK/Beijing/1/94(H9N2)
CK/Hunan/1/12(H9N2)
CK/Shandong/02/10(H9N2)
DK/Hubei/S2227/12(H4N2)
Group 2
97
DK/Guangdong/S1469/10(H4N2)
SW/Guangdong/K4/11(H4N8)
DK/Guangdong/W12/11(H3N2)
DK/Guangdong/S4040/11(H4N2)
DK/Guangodng/S1123/12(H4N2)
GS/Guangdong/S1780/12(H4N2)
Group 3
ML/Korea/GH171/07(H7N7)
91
WD/Korea/SH5-26/08(H4N6)
DK/Hubei/S2213/12(H4N2)
100
DK/Hunan/S2046/11(H4N2)
DK/Jiangxi/S21046/12(H4N2)
DK/Chongqing/S2086/12(H4N8)
DK/Guangxi/GXd-4/11(H1N2)
DK/Victoria/512/07(H7N6)
GS/Hunan/S2466/11(H4N8)
QA/Nanchang/7-026/00(H3N6)
DK/Henan/S1091/10(H4N6)
ML/ZhaLong/88/04(H4N6)
DK/Korea/DY97/07(H4N3)
DK/Henan/S4179/09(H4N6)
DK/Hunan/8-19/09(H4N2)
RSD/Mongolia/2-79/07(H3N8)
DK/Shanghai/28-1/09(H4N2)
CK/Guangdong/S1010/10(H4N8)
DK/Fujian/S2169/12(H4N6)
DK/Nanchang/4-165/00(H4N6)
DK/Hubei/S2114/12(H4N8)
AV/Japan/8KI0148/08(H4N2)
CK/Shandong/S2510/12(H4N2)
DK/Hunan/S1012/09(H4N6)
CB/Mongolia/2-69/07(H10N6)
DK/Zhejiang/S2088/11(H4N6)
DK/Jiangxi/S21055/12(H4N2)
ML/Yan chen/05(H4N6)
DK/Anhui/S4155/09(H4N6)
DK/Anhui/S2193/12(H4N6)
AB/Korea/CN5/09(H6N5)
DK/Jiangsu/S2447/11(H4N6)
GS/Jiangsu/S2433/11(H4N6)
CK/Hunan/S1248/10(H4N6)
CK/Hunan/S1267/10(H4N6)
ML/Sweden/95/05(H7N7)
DK/Jiangxi/S2443/12(H4N6)
DK/Taiwan/WB459/04(H6N5)
DK/Guangxi/S2090/12(H4N6)
DK/Hunan/S11090/12(H4N6)
DK/Hong Kong/T37/97(H11N8)
DK/Jiangxi/S3261/09(H4N2)
DK/Zhejiang/S2235/12(H4N6)
DK/Fujian/S1487/09(H4N3)
0.01
Group 4
Allele A

Fig. S1F

Fig. S1 Phylogenetic analyses of the six internal genes of H4 avian influenza viruses isolated from live poultry markets in China between 2009 and 2012. Phylogenetic trees were generated by using the neighbor-joining method of the MEGA5.0 software package. The regions of nucleotide sequence used for the phylogenetic analyses were: PB2, 37-2289; PB1, 25-2233; PA, 25-2167; NP, 46-1526; M, 26-1007; and NS, 27-859. The phylogenetic trees of PB2 (A), PB1 (B), PA (C), NP (D), M (E), and NS (F) were rooted to A/equine/Prague/1/1956 (H7N7). The colors of the virus names in the phylogenetic trees match with the colors of their HA genes. Abbreviations: AB, aquatic bird; AV, avian; BP, baer's pochard; CB, canvasback; CK, chicken; DK, duck; EN, environment; GS, goose; MD, migratory duck; ML, mallard; PT, pintail; QA, quail; RSD, ruddy shelduck; SBD, spot-billed duck; SW, swine; WB, wild bird; WD, wild duck; WFG, white-fronted goose; WG, wild goose; WS, whooper swan.
